# Gastric Microbiome Diversities in Gastric Cancer Patients from Europe and Asia Mimic the Human Population Structure and Are Partly Driven by Microbiome Quantitative Trait Loci

**DOI:** 10.3390/microorganisms8081196

**Published:** 2020-08-06

**Authors:** Bruno Cavadas, Rui Camacho, Joana C. Ferreira, Rui M. Ferreira, Ceu Figueiredo, Alvis Brazma, Nuno A. Fonseca, Luísa Pereira

**Affiliations:** 1 i3S-Instituto de Investigação e Inovação em Saúde, Universidade do Porto, 4200-135 Porto, Portugal; joanaf@ipatimup.pt (J.C.F.); ruif@ipatimup.pt (R.M.F.); cfigueiredo@ipatimup.pt (C.F.); luisap@ipatimup.pt (L.P.); 2 IPATIMUP—Instituto de Patologia e Imunologia Molecular, Universidade do Porto, 4200-135 Porto, Portugal; 3 ICBAS—Instituto de Ciências Biomédicas Abel Salazar, Universidade do Porto, 4050-313 Porto, Portugal; 4 FEUP-Faculdade de Engenharia, Universidade do Porto, 4200-465 Porto, Portugal; rcamacho@fe.up.pt; 5 INESC TEC—Instituto de Engenharia de Sistemas e Computadores, Tecnologia e Ciência, Universidade do Porto, 4200-465 Porto, Portugal; 6 Faculdade de Medicina, Universidade do Porto, 4200-319 Porto, Portugal; 7 European Molecular Biology Laboratory, European Bioinformatics Institute, EMBL-EBI, Wellcome Trust Genome Campus, Hinxton, Cambridge CB10 1SD, UK; brazma@ebi.ac.uk; 8 CIBIO—Centro de Investigação em Biodiversidade e Recursos Genético, Universidade do Porto, 4485-661 Vairão, Portugal; nuno.fonseca@cibio.up.pt

**Keywords:** gastric microbiome, gastric cancer, European and Asian diversity, biomarkers, microbiome quantitative trait loci, miQTL

## Abstract

The human gastrointestinal tract harbors approximately 100 trillion microorganisms with different microbial compositions across geographic locations. In this work, we used RNASeq data from stomach samples of non-disease (164 individuals from European ancestry) and gastric cancer patients (137 from Europe and Asia) from public databases. Although these data were intended to characterize the human expression profiles, they allowed for a reliable inference of the microbiome composition, as confirmed from measures such as the genus coverage, richness and evenness. The microbiome diversity (weighted UniFrac distances) in gastric cancer mimics host diversity across the world, with European gastric microbiome profiles clustering together, distinct from Asian ones. Despite the confirmed loss of microbiome diversity from a healthy status to a cancer status, the structured profile was still recognized in the disease condition. In concordance with the parallel host-bacteria population structure, we found 16 human loci (non-synonymous variants) in the European-descendent cohorts that were significantly associated with specific genera abundance. These microbiome quantitative trait loci display heterogeneity between population groups, being mainly linked to the immune system or cellular features that may play a role in enabling microbe colonization and inflammation.

## 1. Introduction

Current estimates place the beginning of the cooperative interaction between microbes and their animal hosts in the last 500 million years [1]. Examples of such close interactions in the gastrointestinal tract are Bacteroidaceae and Bifidobacteriaceae, which have been symbionts of primates for over 15 million years [2]. A big increase in normal human microbiome knowledge is being contributed by large scale studies such as the Human Microbiome Project (HMP, led by the United States of America) [3] and MetaHIT (led by the European Union and China) [4]. In the particular case of the largest human microbiome reservoir, the gastrointestinal tract, it harbors approximately 100 trillion microorganisms in a proportion of approximately 1:1 to human cells [5,6], with up to 100 times more genes than the human genome [4,7].

The microbiome composition is dynamic and is affected by factors such as diet, antibiotics, age and ethnicity. By analysing the gut microbiome in stool samples from BaAka pygmies and its neighbor Bantus, and comparing them with samples collected in the United States, Gomez et al. [8] showed that hunter-gatherers harbored an increased abundance of Prevotellaceae, Treponema, and Clostridiaceae, associated with a profile of increased virulence, and amino acid and vitamin metabolism. The gut microbiome of Bantu was dominated by Firmicutes and displayed western-like features, such as an increased abundance of predictive carbohydrates and xenobiotic metabolic pathways. Nevertheless, the two African groups were more similar to each other in terms of their gut microbiome diversity than either group against the USA cohort. Additionally, there was a loss of (alpha) diversity in the industrialized population, mainly due to the loss of the bacterial taxa involved in fiber processing, coupled with a gain of lineages associated with the consumption of agricultural products. The gut microbiome of the Danish is generally enriched with the phylum Firmicutes, including *Oenococcus* and other lactic acid bacteria [9], whereas that of the Chinese has greater abundance of Proteobacteria. A genome-wide association study of the gut microbiota in 1812 Germans [10] identified significant associations for the overall microbial variation and individual taxa at multiple loci, especially with the *VDR* (vitamin D receptor) gene, several disease susceptibility genes and sterol metabolism pathway components. The authors estimate that non-genetic and genetic factors account each for 10% of gut microbiota variations. A recent study conducted in a large cohort of Flemish and German individuals lead to the identification of further genetic associations involving multiple microbial traits, including that between a single nucleotide polymorphism (SNP) on the *RAPGEF1* gene, which is involved in the regulation of the gastrointestinal tract’s physiology, and *Ruminococcus* [11].

Microbiome homeostasis is critical for human health. Changes in the microbiome composition, also known as dysbiosis, have now been associated with several diseases, such as obesity [12], atherosclerosis [13], inflammatory bowel disease [14], Alzheimer’s [15], and cancer [16]. In the gastric cancer setting, studies that characterized the gastric microbiome found complex bacterial communities dominated by different species with a low abundance of *H. pylori* in the stomach of cancer patients [17,18,19,20]. Divergent microbial profiles associated with cancer were described in these studies, which may be explained in part by the different technical approaches, namely if the original sample was obtained from a gastric juice or mucosal specimen, and also due to the diverse geographic origins of the patients studied.

The Cancer Genome Atlas (TCGA) has collected the largest adenocarcinoma cohort of patients from several countries in Europe (Germany, Poland, Ukraine and Russia), Asia (Vietnam and South Korea), Oceania (Australia) and America (the United States (USA), Canada and Brazil), and makes freely available whole genome/exome sequences (WGS/WES) and transcriptomes [21]. While the number of WGS-typed samples is limited to around 50, RNASeq has been performed in all cancer samples and in around 30 samples from adjacent normal tissue. It has been shown that it is possible to identify and quantify non-human sequenced fragments (including bacteria and viruses) from human-centred WGS [22,23] and RNASeq [23,24]. Another interesting public database is the Genotype-Tissue Expression (GTEx), which provides RNASeq of several tissues from deceased non-cancer patients [25] in the United States, mostly of European descent, including approximately 200 gastric samples. The GTEx has been intensively used to infer expression quantitative trait loci (eQTL) in the various human tissues. Currently, these two databases are the richest in terms of the geographical origin of samples, completeness of human omic characterization, clinical classification, and direct gastric tissue sampling. Nevertheless, the microbiome was not profiled in these samples. In this sense, the inference of the microbial community in the TCGA and GTEx datasets would provide a comprehensive investigation on the role of host diversity in shaping the microbiome dynamics.

In this work, we departed from the TCGA and GTEx RNASeq datasets to investigate and to infer the bacterial microbiome in cancerous and non-cancerous gastric tissues. Furthermore, we evaluated the microbiome variation attending to the human population structure and searched for microbiome quantitative trait loci (miQTL).

## 2. Materials and Methods 

### 2.1. Samples

Gastric adenocarcinoma RNASeq raw reads, unmapped to the GRCh38 human genome encompassing 375 tumors and 27 matched normal tissues, were obtained from aligned RNASeq data on the TCGA Genomic Data Commons repository (https://gdc.cancer.gov/). For these samples, information regarding their origin, sex, age, ethnicity, pathogenic (type and location of tumor, pathogenicity scoring), and clinical information (treatment and vital status) was available. For this tissue, the geographic collection of samples spanned 11 countries (Australia, Brazil, Canada, Germany, Moldova, Poland, South Korea, Ukraine, the United Kingdom, the United States and Vietnam). Following the same rationale, we have also inferred the microbiome profile of stomach samples of non-disease individuals collected in the United States through the analysis of RNASeq data from the GTEx database [25]. From the 202 stomach GTEx samples, we excluded samples with an ethnicity other than “white” and with clinical evidence of gastritis, acid reflux or other gastric pathologies, leaving a total of 164 samples. The distribution of the samples used in this work is summarized in Supplementary Table S1. The original material was frozen tissue, collected under careful standard operating procedures available at the consortia websites (https://brd.nci.nih.gov/brd/sop-compendium/show/701; https://biospecimens.cancer.gov/resources/sops/default.asp).

This study was carried in accordance with the recommended data access guidelines from the TCGA and GTEx datasets. We received administrative permission for the download of restricted-access data from stomach cancer patients (TCGA) and patients with normal stomachs from deceased individuals (GTEx). Additional approval by the local Ethics Committee was not required.

### 2.2. Microbiome Inference

A total of 900 bacterial whole genomes (one representative strain per species; Supplementary Table S2) were collected from NCBI following the species identified by the HMP [3] and MetaHit [9] projects in the gastrointestinal tract and complemented the species identified in the works of Rajilić-Stojanović and de Vos [26] and Ferreira et al. [20].

The pipeline used in this manuscript to characterize and quantify the bacterial species is described in detail elsewhere [27], and summed up in Supplementary Figure S1. Briefly, QmihR began by trimming reads using Trimmomatic (v0.36) [28], checking if: (1) the average Phred of two consecutive reads was below 20, and (2) the read length was smaller than 40 bases. These reads were then aligned, by global alignment, against the bacterial reference database with Bowtie2 (v2.2.7) [29] and the quantification of bacterial genera was performed through RSEM (v1.2.29) [30] for the probabilistic assignment of multi-mapping reads. The pipeline aggregates the counts of the mapped reads in the various genes within a species to produce counts of reads aligned per species. After analyses, we limited counting reads to those mapped to bacterial rRNA genes. Reads belonging to the same genera were combined and normalized by the library size for the total mapped reads against the bacterial reference database.

### 2.3. Checking for Genus Coverage, Richness and Evenness

Estimations of the genus coverage, richness, evenness, and diversity were calculated in R using the Phyloseq package (v1.23.1) [31]. The alpha diversity was determined by the Shannon index. Good’s coverage calculated an estimator of the sample coverage of a community. The Chao1 estimator returned the genus richness based on a vector or matrix of abundance data, reflecting the proportion of singletons and doubletons in the dataset. The evenness reflects the closeness in the number of genera, corresponding to Shannon’s index divided by the number of genera.

### 2.4. Phylogenetic Analyses

The phylogenetic information for the genera present in our samples was retrieved (on the 20th January 2019) from the Time Tree website (http://www.timetree.org/). This information was used to calculate unweighted and weighted UniFrac distances. The UniFrac distances are beta measures (the mean genera diversity among samples) that take into account the similarities and differences among genera and including the weighted or unweighted genera abundances in calculations [32]. The Bray–Curtis dissimilarity, another distance-based method, takes into account the counts at each site. The unrooted neighbor-joining tree was used to represent the weighted UniFrac distances between TCGA population samples. A permutation multivariate analysis (1000 permutations) of variance (PERMANOVA; [33]) of the weighted UniFrac distances (to reflect the phylogenetic diversity between genera) was also performed and plotted in a non-metric multidimensional scale (nMDS). The null hypothesis tested by PERMANOVA was that the centroids of each group are equivalent, under the assumption of exchangeability of the samples among groups.

A t-distributed stochastic neighbor embedding (t-SNE) analysis based on the normalized values was estimated to check for the batch influence on the beta-diversity. This analysis was conducted in R using Rtsne package (v0.15) [34].

### 2.5. Evaluating Microbiota Composition in Gastric Tumorigenesis

A differential expression was applied to evaluate the changes in the microbiome abundance (for genera and phyla) by using the DESeq2 package (v1.24.0) [35]. The heatmap reflecting these changes was obtained in R using the gplots (v3.0.1.1) package.

### 2.6. Host-Genome and Microbiome Associations

**Figure 1:**
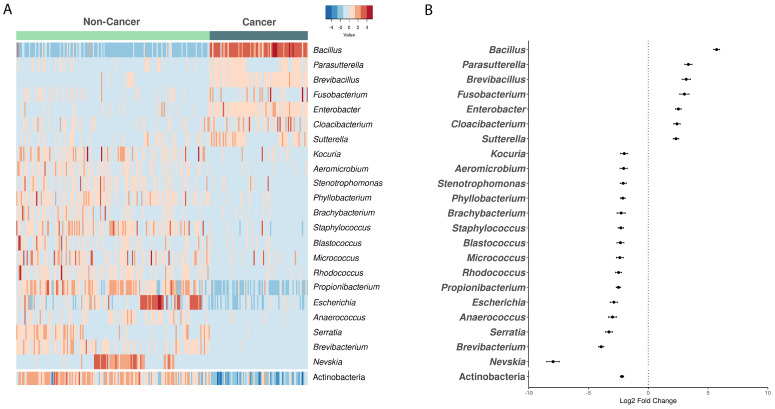
Microbiota composition in gastric cancer and non-cancerous samples. (**A**) Heatmap of statistically significant differentiated genera and phyla abundance when comparing the control Genotype-Tissue Expression (GTEx) non-cancer gastric microbiome with the European ancestry (Europe and USA) The Cancer Genome Atlas (TCGA) cancer samples. (**B**) Log2 fold change of the statistically significant differentiated genera and phyla between the non-cancer (negative axis) and cancer (positive axis) cohorts.

The association of host germline genotypes with microbiome genera abundance was tested in the TCGA and GTEx datasets. The tested genera were the ones listed in Figure 1, which displayed frequencies above 1% in at least one population. Genera abundance was further normalized by an arc-sin transformation to obtain normality. These miQTLs were identified by employing Matrix eQTL software (v2.2) [36]. Host germline variants, common to both datasets, were limited to the MAF > 0.05 (minimum allele frequency), non-synonymous type and were in a Hardy–Weinberg equilibrium (*p*-value > 1 × 10^−5^). The country of origin, sex, age, sequencing center and year of sample shipment were considered as confounding variables in all analyses. The *p*-values were subsequently corrected for multiple testing using Storey’s *q*-value method [37]. To check the *F*_ST_ distances between the human population groups for these variants, we used the African, Asian and European populations from the 1000 Genomes project [38].

## 3. Results

### 3.1. Quality Control of RNASeq Data for Microbiome Inference

After retrieving bacterial reads from the transcriptomic data of the TCGA and GTEx and, since batch effects in TCGA samples have been described [39,40], an initial quality control of the data was performed. A characterization based on diversity revealed that samples in batches with a year of shipment between 2010 and 2012 had a statistically significant (*p* < 2.2 × 10^−16^) higher diversity than the batches from 2013 and 2014 (median Shannon diversity of 2.92 (2010–2012); 1.31 (2013 to 2014), Supplementary Figure S2A). A detailed inspection of the relative abundance of bacteria per sample showed that some samples had an almost monophyletic microbiome. The most extreme case was found in the samples which were shipped in 2013 and 2014, which contained almost exclusively *Pseudomonas* (128 samples, mean relative abundance of 95% (Supplementary Table S3)). The samples shipped in 2010, 2011 and 2012 had a more variable microbiome (247 samples in total; with a 20% mean relative abundance of *Pseudomonas*), mostly made of three similarly frequent genera and many less frequent ones. These results led us to conclude that the 2013 and 2014 batches were possibly contaminated by *Pseudomonas* (Supplementary Figure S2B) and were thus removed from further analyses. No discernible differences between batches in the GTEx samples were observed (data not shown). Even though the alpha diversity analysis showed similar values of diversity for the 2010, 2011 and 2012 batches, we further assessed the batch-specific enrichment of genera. The results showed (Supplementary Figure S2C) that the 2010 and 2011 batches cluster together into two different sub-clusters, while the 2012 samples showed a more homogeneous distribution. Given the small sample sizes of the 2010 and 2011 batches (*n* = 37 and *n*= 73, respectively) comparatively to the 2012 batch (*n* = 137), we conservatively limited further analyses to the TCGA 2012 cohort (largely European and East Asian samples).

We then checked where in the bacterial genome these reads were mapping to, observing that 81.1% of all the aligned RNASeq reads mapped to the ribosomal RNA (rRNA) genes. On average, 9356 and 7171 reads in the TCGA and GTEx samples, respectively, mapped to rRNA genes with the following equivalent distributions (*p* = 0.74): 39.66% and 36.47% on 16S rRNA; 60.12% and 63.17% on 23S rRNA; 0.22% and 0.36% on 5S rRNA. To avoid biases due to differentially expressed protein coding-genes, we limited all further analyses to bacterial rRNA-mapped reads.

### 3.2. Microbiome Composition in Cancer vs. Non-Cancer Samples from European Ancestry

Previous investigations using 16S rRNA-based methods have disclosed differences in the gastric microbiome between patients with and without cancer [18,19,20], mainly in the decreased microbiome diversity in patients with cancer status. Therefore, we inferred the gastric microbiome from the TCGA (cancer cohort) and GTEx (non-cancer cohort) datasets. However, since the GTEx dataset originates from USA individuals, who are mostly of European ancestry (*n* = 164), we limited this comparison to the TCGA samples from European and USA (*n*= 83) origins.

We assessed the microbial diversity in our inferred data, through metrics such as Good’s coverage, evenness, alpha diversity (Shannon index) and Chao1 estimator. The median estimated coverage was above 97% in each group (Supplementary Figure S3). The bacterial richness, evenness, and Shannon diversity were significantly lower in cancer than in non-cancer samples (*p* = 1.1 × 10^−9^, *p* < 2.2 × 10^−16^, and *p* < 2.2 × 10^−16^, respectively; Supplementary Figure S3).

To evaluate whether there were differences in taxa that could explain the variation in diversity, we performed a differential analysis of taxa. The results showed that some genera reached statistically significant differences above two log fold changes between non-cancer and cancer cohorts: *Bacillus* (*p* = 1.5 × 10^−113^), *Parasutterella* (*p* = 7.5 × 10^−27^), *Brevibacillus* (*p* = 4.9 × 10^−18^), *Fusobacterium* (*p* = 9.4 × 10^−14^), *Enterobacter* (*p* = 8.6 × 10^−24^), *Cloacibacterium* (*p* = 1.48 × 10^−17^) and *Suterella* (*p* = 2.3 × 10^−21^) were enriched in the microbiome of cancer samples, whereas, *Kocuria* (*p* = 2.6 × 10^−10^), *Aeromicrobium* (*p* = 1.0 × 10^−10^), *Stenotrophomonas* (*p* = 6.0 × 10^−15^), *Phyllobacterium* (*p* = 8.6 × 10^−24^), *Brachybacterium* (*p* = 5.4 × 10^−10^), *Staphylococcus* (*p* = 2.5 × 10^−22^), *Blastococcus* (*p* = 5.6 × 10^−14^), *Micrococcus* (*p* = 3.2 × 10^−15^), *Rhodococcus* (*p* = 3.1 × 10^−20^), *Propionibacterium* (*p* = 1.5 × 10^−38^), *Escherichia* (*p* = 7.7 × 10^−19^), *Anaerococcus* (*p* = 3.0 × 10^−16^), *Serratia* (*p* = 6.9 × 10^−27^), *Brevibacterium* (*p* = 4.9 × 10^−18^) and *Nevskia* (*p* = 1.2 × 10^−47^) were significantly enriched in non-cancer samples (Figure 1A,B). There were no differences in the relative abundance of these genera between the four cancer stages (AJCC pathological tumor staging; Supplementary Figure S4). The differences in genera abundance led to phylum Actinobacteria (*p* = 9.4 × 10^−51^) being more abundant (with statistical significance) in the non-cancer cohort (Figure 1A,B).

### 3.3. Microbiome Profiling in Function of Host Geographic Origin

Taking into consideration that, for the gastric adenocarcinoma, the TCGA cohort includes samples collected from multiple geographic locations, we leveraged this information to evaluate whether the microbiome varied between the main human ancestral backgrounds. 

**Figure 2:**
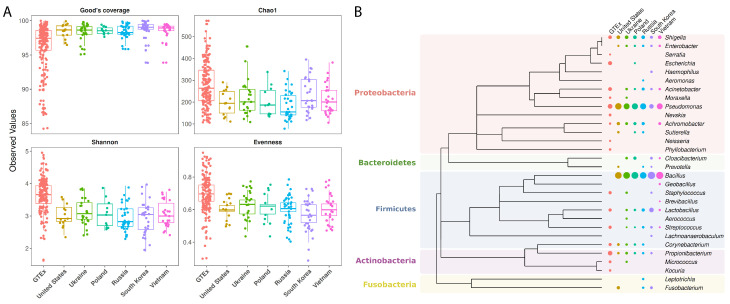
Alpha diversity and phylogenetic analysis of the gastric microbiome. (**A**) Good’s coverage, Chao1, Shannon and evenness in the inferred microbiome patterns. (**B**) Phylogenetic tree and respective abundance of genera found in the gastric microbiome (only genera that passed a mean threshold of 1% in at least one population are displayed in the graph). Samples are from non-cancer individuals from the GTEx dataset (United States *n* = 164, in brink pink) and cancer patients from the TCGA 2012 batch (United States *n* = 14 in gold; Ukraine *n* = 24 in limeade, Poland *n* = 11 in Caribbean green; Russia *n* = 34 in cerulean; South Korea *n* = 28 in purple and Vietnam *n* = 26 in pink).

We first checked if the RNASeq-based inference had enough resolution to reliably characterize the microbiome diversity from all the geographic locations available. As can be seen in Figure 2A, Good’s coverage per sample was always higher than 84%, with 57% of samples having this estimate > 98%, and 25% of samples having > 99%. Chao1 estimated a median of 196 and 264 genera for the various populations in the TCGA and GTEx datasets, respectively. Overall, within the TCGA cancer cohort, the bacterial richness and evenness were similar in all populations, and the Shannon index was not significantly different between continents. 

We next evaluated if the microbiome had a different signature across continents. In agreement with previous descriptions of the gastric microbiome [41,42], genera found in the GTEx and TCGA stomach samples belonged mainly to Proteobacteria, Firmicutes, Actinobacteria, Bacteroidetes and Fusobacteria phyla (Figure 2B). Firmicutes was the dominant phylum observed in Asia, while Proteobacteria was dominant in Europe (53.6% and 39.7%, and 34.0% and 47.3%, respectively). These differences between continents were statistically significant (*p* = 1.9 × 10^−6^ and *p* = 2.9 × 10^−5^, respectively). No statistically significant differences were observed between the remaining phyla.

Within the cancer cohort, *Bacillus* was the dominant genus in European and East Asian samples, despite the relative abundance of this genus being significantly higher in the latter (30.4% and 38.1%, respectively; *p* = 4.1 × 10^−3^). *Bacillus* was followed by *Pseudomonas* (24.2% and 15.9%; *p* = 7.9 × 10^−5^*)*, *Shigella* (5.4% and 4.2%; *p* = 4.9 × 10^−3^)*,* and *Lactobacillus* (2.3% and 6.4%; *p* = 0.1), while *Helicobacter* was vestigial (lower than 1%).

**Figure 3:**
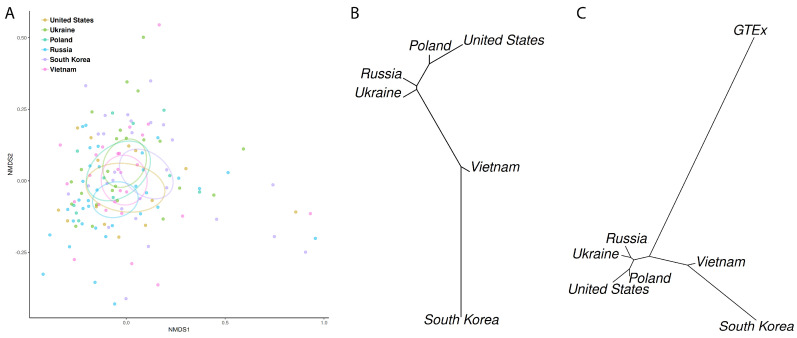
Gastric cancer microbiome profiling in function of the host geographical origin. (**A**) non-metric multidimensional scale (nMDS) plot for the weighted UniFrac distances and PERMANOVA analysis (stress = 0.17; r2 = 0.05; *p*~0.054) between the geographic groups in the cancer cohort (same colors as in Figure 1). (**B**) Neighbor-joining tree of the mean weighted UniFrac distances between the geographic groups in the cancer cohort. (**C**) Neighbor-joining tree of the mean weighted UniFrac distances between the geographic groups in the cancer and non-cancer cohorts.

Still within the cancer cohort, but considering the genera per country of origin, the USA had a high relative abundance of *Fusobacterium* (4.0%) and a low relative abundance of *Lactobacillus* (0.3%), while the opposite was observed in the central-eastern group of Ukraine, Poland and Russia (1.0% and 1.5% in Ukraine, 0.3% and 1.7% in Poland, and 1.6% and 3.9% in Russia). *Bacillus* accounted for 28% of the reads in samples from Russia. In Asia, the samples from South Korean and Vietnamese had a high relative abundance of *Bacillus* (40.0% and 37.1%, respectively) and *Pseudomonas* (10.0% and 22.1%, respectively), but while South Korea had more *Lactobacillus* (11.0%) and *Fusobacterium* (2.6%) and less *Achromobacter* (0.8%), Vietnam had the opposite trend (1.4%, 0.4% and 2.7%, respectively). These profiles are statistically different for the comparisons between European and East Asian groups, as evaluated in the nMDS plot for the weighted UniFrac distances and PERMANOVA analysis (stress = 0.17; r2 = 0.05; *p* = 0.054; Figure 3A): USA vs. South Korea, *p* = 0.048; Poland vs. South Korea, *p* = 0.024; South Korea vs. Vietnam, *p* = 0.006. By displaying the mean weighted UniFrac distances between groups in a neighbor-joining tree (Figure 3B), it was observed that the distances between the microbiomes of cancer samples from different geographic locations reflect the pattern of the genetic distances between main human populations: European populations clustering together, and East Asian populations in another branch. Thus, the population structure was observed in the microbiome of tumor samples. The microbiome of the non-cancerous samples GTEx (mainly European-Americans) was closer to the European tumor cohort (Figure 3C), reflecting a common human ancestry.

### 3.4. Identification of Host Genetic Variation Associated with the Microbiome

Table 1 summarizes the 16 significant miQTLs, 12 in the TCGA European and 4 in the non-diseased gastric GTEx cohort. No miQTLs were observed at a q-value lower than 0.1 in the TCGA Asian cohort, most probably due to the low power of the limited Asian sample size. Some of the European miQTLs identified in this study already had a known interaction with the microbiome. The association between the *DPH1* gene and *Corynebacterium* was described before—this gene encodes an enzyme involved in the secondary transformation (diphtamidation) of histidines in elongation factor 2 (*EEF2*), which is a target of the diphtheria toxin secreted by *Corynebacterium diphtheriae* [43]. The association between the *ZC3H12D* gene and *Lactobacillus* and *Fusobacterium* may be related with the regulation of the inflammatory response [44]. Other miQTLs found in this study have been previously linked to microbial features, although not directly associated with specific genera. The *TRIM31* gene was found to inhibit invasive bacteria by the induction of an autolysosome. The downregulation of this gene by the human cytomegalovirus results in the hyperproliferation of invasive bacteria [45]. Multiple kinesin motors, including *KIF24*, are required for the formation of *Salmonella*-induced filaments during infection by *Salmonella enterica* [46]. The other miQTL are novel and need a more extensive investigation.

**Figure 4:**
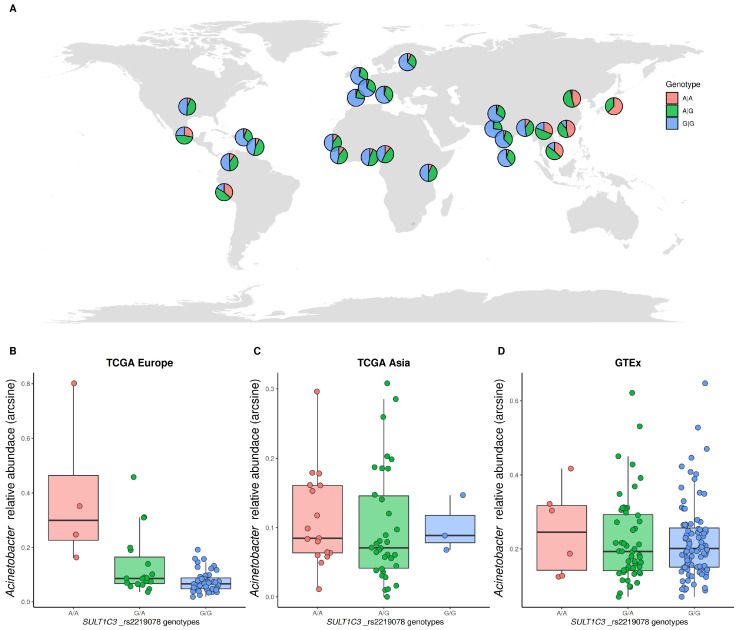
Example of an identified microbiome quantitative trait locus. (**A**) Worldwide genotype frequencies of rs2219078 SNP located in the *SULT1C3* gene for 1000 Genomes project populations. (**B**) *Acinetobacter* relative abundances of the rs2219078 genotypes in the TCGA European cohort. (**C**) The TCGA East Asian cohort. (**D**) The GTEx European-descendent cohort.

Interestingly, most of the miQTLs displayed heterogeneity between population groups, thus explaining in part the microbiome heterogeneity related with human ancestry. The heterogeneity was inferred from the *F*_ST_ distances between population groups based on the genotype frequencies from the 1000 Genomes project [38] (Table 1). All miQTLs except rs1014867-*FAT4* showed significant genetic distances between at least two population groups, indicating that these SNPs are very heterogeneous. An illustrative example of this is the association between the rs2219078 SNP located in the *SULT1C3* gene with *Acinetobacter* (Figure 4), with allele A being positively associated with a higher frequency of this bacteria (statistically significant in the TCGA European cohort (False discovery rate (FDR) adjusted *p*-value of 0.024), and following the same tendency in the TCGA East Asian and GTEx European cohorts). Homozygous AA are relatively rare in Europe (4.2%) and Africa (8.5%), but very frequent in East Asia (45.0%). This SNP was also identified as an eQTL (expression quantitative trait loci) in the stomach (allele A associated with a higher gene expression) by the GTEx project, and the *SULT1C3* gene plays a role in sulfotransferase activity, being involved in the biotransformation of xenobiotics [47]. Curiously, several *Acinetobacter* strains are used in environmental and biotechnological applications for the biodegradation, leaching and removal of several organic and inorganic compounds [48]. The associated human gene and bacteria may have an additive function in the metabolism of xenobiotics.

## 4. Discussion

In this work, we have indirectly characterized the gastric microbiome of gastric cancer patients and deceased non-cancer individuals from RNASeq data obtained from the TCGA and GTEx projects, respectively. We demonstrated that the microbiome inference was reliable, even and rich for genera coverage, after a careful evaluation of batch-contamination by *Pseudomonas* (extending the contamination issues already noticed by [40]) and batch-specific microbiome compositions. Due to the indirect classification from human-based RNASeq data, some inherent limitations were unavoidable. The samples were collected and processed using human specific protocols lacking microbial DNA/RNA-free reagents, microbiome-related negative controls, as well as microbial RNA-specific enrichment steps. RNASeq in both these projects was enriched by poly-A selection. Polyadenylation was thought to be scarcely present in bacteria [49]. However, in contrast to eukaryotic cells, polyadenylation is known to not be restricted to the mRNA in bacteria, with 5S [50], 16S and 23S rRNA also being polyadenylated [51]. Given that these bacterial rRNAs were the most abundant transcripts found in this study, it is not surprising that we were able to identify abundant bacterial reads in the non-human RNASeq databases. 

The microbiome inference in large well-characterized cohorts of samples from diverse populations, directly in the stomach tissue, confirmed known evidence and revealed new findings. Specifically, our results confirmed the loss of microbiome diversity in gastric cancer patients [19,20] with a recognizable structured microbiome profile in the disease condition, and uncovered novel relationships between bacteria and host ancestry. Within the European-descendant background, a low microbial diversity in cancer cases was accompanied by a decrease in the abundance of *Rhodococcus*, *Phyllobacterium* and *Staphylococcus*, which was counterbalanced by the increase in *Bacillus*, *Enterobacter*, *Fusobacterium* and *Sutterella*. At the phylum level, a statistically significant difference in Actinobacteria was observed. The low frequency of non-cancer patients of Asian ancestry in GTEx did not help ascertain whose bacteria could be candidate cancer biomarkers in this ancestry. Using a non-metric multidimensional scaling analysis with the UniFrac distance, we verified the significant differences between the European and East Asian groups of patients, which reinforces the role of geographic origins in shaping the microbiome composition. In fact, these differences observed between continents may help to explain differences in the identification of bacterial species associated with gastric cancer in the various studies [17,18,19,20].

This mimicking of the microbiome diversity in gastric cancer and host diversity across the world is, in part, driven by the associations between microbe genera with human SNPs that are heterogeneous between African, European and Asian populations. Many of these non-synonymous SNPs are located in genes that play a role in the immune response. Our major observations of microbiome structure across the globe, driven by the microbiome quantitative trait loci, reinforce the importance of the microbiome–host binomial. This knowledge is essential for the identification of predictive biomarkers, by which the host and microbe genetic factors must be taken into account to predict microbiome-related conditions. Therefore, we propose that the results of microbiome studies should be interpreted considering the geographic origin and population genetic background of the host.

## Figures and Tables

**Table 1:** Statistically significant (*q*-value < 0.15) microbiome quantitative trait loci (miQTLs) in the TCGA-European and GTEx cohorts. No miQTLs were identified in the Asian cohort.

	**SNP**	**Genus**	**GeneID**	***p*** **-Value**	***q*** **-Value**	**Genotype Frequency**			**Fst (*p*-Values)**		
						**MAF_EUR**	**MAF_EAS**	**MAF_AFR**	**EUR_AFR**	**EUR_EAS**	**EAS_AFR**
TCGA-EUROPE	rs1131600	*Corynebacterium*	*DPH1*	7.97104 × 10^−8^	0.01501	G|G: 0.028 A|G: 0.252 A|A: 0.720	G|G: 0.004 A|G: 0.105 A|A: 0.891	A|G: 0.020 A|A: 0.980	0.14323 (0.00000 ± 0.0000)	0.04831 (0.00000 ± 0.0000)	0.0359 (0.00000 ± 0.0000)
TCGA-EUROPE	rs61997220	*Lactobacillus*	*ZC3H12D*	3.07635 × 10^−7^	0.02414	T|T: 0.907 C|T: 0.093	T|T: 1.000	T|T: 0.995 C|T: 0.005	0.04507 (0.00000 ± 0.0000)	0.04582 (0.00000 ± 0.0000)	0.00113 (0.25978 + 0.0048)
TCGA-EUROPE	rs9262143	*Acinetobacter*	*PPP1R18*	5.68970 × 10^−7^	0.02414	C|C: 0.845 C|T: 0.151 T|T: 0.004	C|C: 1.000	C|C: 0.995 C|T: 0.005	0.08232 (0.00000 ± 0.0000)	0.07868 (0.00000 ± 0.0000)	0.00113 (0.26255 ± 0.0046)
TCGA-EUROPE	rs17350674	*Cloacibacterium*	*KIF24*	5.97391 × 10^−7^	0.02414	C|C: 0.614 A|A: 0.050 A|C: 0.336	C|C: 0.988 A|A: 0.002 A|C: 0.010	C|C: 0.980 A|C: 0.020	0.21379 (0.00000 ± 0.0000)	0.19977 (0.00000 ± 0.0000)	0 (0.52688 ± 0.0052)
TCGA-EUROPE	rs2219078	*Acinetobacter*	*SULT1C3*	6.40940 × 10^−7^	0.02414	G|G: 0.658 A|G: 0.300 A|A: 0.042	G|G: 0.097 A|G: 0.452 A|A: 0.450	G|G: 0.472 A|G: 0.443 A|A: 0.085	0.03291 (0.00000 ± 0.0000)	0.38529 (0.00000 ± 0.0000)	0.24086 (0.00000 ± 0.0000)
TCGA-EUROPE	rs2523989	*Acinetobacter*	*TRIM31*	1.03982 × 10^−6^	0.03263	C|C: 0.775 C|T: 0.209 T|T: 0.016	C|C: 0.905 C|T: 0.093 T|T: 0.002	C|C: 0.903 C|T: 0.095 T|T: 0.002	0.03296 (0.00000 ± 0.0000)	0.03172 (0.00000 ± 0.0000)	0 (0.99990 ± 0.0000)
TCGA-EUROPE	rs7198494	*Achromobacter*	*C16orf46*	1.75767 × 10^−6^	0.04728	A|A: 0.648 A|G: 0.294 G|G: 0.058	A|A: 0.962 A|G: 0.036 G|G: 0.002	A|A: 0.526 A|G: 0.389 G|G: 0.085	0.01389 (0.00010 ± 0.0001)	0.15724 (0.00000 ± 0.0000)	0.21452 (0.00000 ± 0.0000)
TCGA-EUROPE	rs61997220	*Fusobacterium*	*ZC3H12D*	2.66174 × 10^−6^	0.05857	T|T: 0.907 C|T: 0.093	T|T: 1.000	T|T: 0.995 C|T: 0.005	0.04507 (0.00000 ± 0.0000)	0.04582 (0.00000 ± 0.0000)	0.00113 (0.25978 ± 0.0048)
TCGA-EUROPE	rs62572859	*Lactobacillus*	*C9orf129*	2.79953 × 10^−6^	0.05857	C|C: 0.750 C|T: 0.221 T|T: 0.030	C|C: 0.919 C|T: 0.081	C|C: 0.433 C|T: 0.475 T|T: 0.092	0.09115 (0.00000 ± 0.0000)	0.05751 (0.00000 ± 0.0000)	0.22638 (0.00000 ± 0.0000)
TCGA-EUROPE	rs1014867	*Lactobacillus*	*FAT4*	5.53680 × 10^−6^	0.09807	C|C: 0.895 C|T: 0.103 T|T: 0.002	C|C: 0.885 C|T: 0.109 T|T: 0.006	C|C: 0.865 C|T: 0.126 T|T: 0.009	0.00188 (0.09425 ± 0.0026)	0 (0.56796 ± 0.0055)	0.00016 (0.33403 ± 0.0052)
TCGA-EUROPE	rs1782360	*Lactobacillus*	*LRBA*	6.08310 × 10^−6^	0.09807	G|G: 0.855 C|C: 0.012 C|G: 0.133	G|G: 0.754 C|C: 0.022 C|G: 0.224	G|G: 0.495 C|C: 0.077 C|G: 0.428	0.13144 (0.00000 ± 0.0000)	0.01505 (0.00040 ± 0.0002)	0.06804 (0.00000 ± 0.0000)
TCGA-EUROPE	rs4963198	*Corynebacterium*	LRRC56	6.84488 × 10^−6^	0.09914	G|G: 0.109 A|A: 0.437 A|G: 0.453	G|G: 0.022 A|A: 0.768 A|G: 0.210	G|G: 0.319 A|A: 0.194 A|G: 0.487	0.09743 (0.00000 ± 0.0000)	0.11482 (0.00000 ± 0.0000)	0.33553 (0.00000 ± 0.0000)
GTEx	rs61733127	*Streptococcus*	*PHLPP2*	3.22625 × 10^−11^	0.00001	G|G: 0.024 A|G: 0.249 A|A: 0.728	G|G: 0.012 A|G: 0.109 A|A: 0.879	A|G: 0.056 A|A: 0.944	0.09268 (0.00000 ± 0.0000)	0.03325 (0.00000 ± 0.0000)	0.01635 (0.00000 ± 0.0000)
GTEx	rs74344827	*Streptococcus*	*TAT*	9.87962 × 10^−8^	0.01160	G|G: 0.700 A|G: 0.274 A|A: 0.026	G|G: 0.905 A|G: 0.087 A|A: 0.008	G|G: 0.663 A|G: 0.309 A|A: 0.029	0.00052 (0.22265 ± 0.0035)	0.06188 (0.00000 ± 0.0000)	0.07456 (0.00000 ± 0.0000)
GTEx	rs73229817	*Corynebacterium*	*PDLIM2*	2.60383 × 10^−7^	0.02039	C|C: 0.901 C|T: 0.097 T|T: 0.002	C|C: 1.000	C|C: 0.998 C|T: 0.002	0.05466 (0.00000 ± 0.0000)	0.0498 (0.00000 ± 0.0000)	0 (0.99990 ± 0.0000)
GTEx	rs12807209	*Nevskia*	*MUC6*	5.79755 × 10^−7^	0.03405	G|G: 0.998 C|G: 0.002	G|G: 1.000	G|G: 0.750 C|C: 0.018 C|G: 0.231	0.11679 (0.00000 ± 0.0000)	0 (0.50579 ± 0.0054)	0.11913 (0.00000 ± 0.0000)

## Supplementary Materials

The following supplementary materials are available online at https://www.mdpi.com/2076-2607/8/8/1196/s1, Table S1: Distribution of samples used in this study by cohort, date of collection or DNA extraction (GTEx) and population. Table S2: List of bacterial species used in this study. Figure S1: Schematic representation of the QmihR pipeline. Figure S2: Quality control assessment of the TCGA gastric adenocarcinoma samples based on the raw bacterial counts and alpha diversity (Shannon). Table S3: Percentage of abundance per sample of *Helicobacter* and of genera that reach a value of 90% (*Escherichia*, *Lactobacillus* and *Pseudomonas*), with information on the country of origin and year of shipment. Figure S3: Genus richness in the inferred microbiome patterns between the GTEx non-cancer samples and the TCGA cancer samples from the sample ancestral background (European and United states). Figure S4: Violin plot representation of the relative abundance of the statistically significant differentiated genera in the four cancer stages (AJCC pathological tumor stating) and GTEx non-cancer dataset.
