# Supplementary material for: Gastric Microbiome Diversities in Gastric Cancer Patients from Europe and Asia Mimic the Human Population Structure and Are Partly Driven by Microbiome Quantitative Trait Loci
Source: Microorganisms. 2020 Aug 6;8(8):1196. doi: 10.3390/microorganisms8081196 (PMC7463948; doi:10.3390/microorganisms8081196)
Supplement: Supplementary file 1 [file microorganisms-08-01196-s001.pdf]

Supplementary file

Supplementary Table S1. Distribution of samples used in this study by cohort, date of collection or DNA extraction (GTEx) and population.

Cohort	Date of collection or DNA extraction	Population		
		European	East Asian	Other
TCGA	2010	37	0	0
GTEx		0	0	0
TCGA	2011	66	7	0
GTEx		22	0	0
TCGA	2012	83	54	0
GTEx		54	0	0
TCGA	2013	36	0	4
GTEx		76	0	0
TCGA	2014	15	4	69
GTEx		12	0	0

Supplementary Table S2. List of bacterial species used in this study based on the species identified by the Human Microbiome [1] and MetaHit [2] projects complemented by species identified by Rajilić-Stojanović and de Vos [3]. Additional species appearing in disease conditions identified by Ferreira et al were also added [4]. One representative strain was chosen for each species.

**Species Name**	**RefSeq assembly accession**	**Species Name**	**RefSeq assembly accession**
Abiotrophia defectiva ATCC 49176	GCF_000160075.2	*Geobacillus stearothermophilus DSM 458*	GCF_002300135.1
Absiella dolichum DSM 3991	GCF_000154285.1	*Geobacillus vulcani PSS1*	GCF_000733845.1
Acetanaerobacterium elongatum CGMCC 1.5012	GCF_900103835.1	*Georgenia muralis DSM 14418*	GCF_003814705.1
Acetoanaerobium sticklandii DSM 519	GCF_000196455.1	*Gordonia rubripertincta CWB2*	GCF_003568625.1
Acetomicrobium hydrogeniformans ATCC BAA-1850	GCF_000160455.2	*Gordonia terrae 3612*	GCF_001698225.1
Achromobacter denitrificans	GCF_003812265.1	*Gordonibacter pamelaeae 7-10-1-b*	GCF_000210055.1
Achromobacter piechaudii ATCC 43553	GCF_000164035.1	*Granulicatella adiacens ATCC 49175*	GCF_000160675.1
Achromobacter sp. MFA1 R4	GCF_900156745.1	*Granulicatella elegans ATCC 700633*	GCF_000162475.2
Achromobacter xylosoxidans NCTC10807	GCF_001457475.1	*Grimontia hollisae ATCC 33564*	GCF_001558255.2
Acidaminococcus fermentans DSM 20731	GCF_000025305.1	*Haemophilus haemolyticus M19346*	GCF_003352385.1
Acidaminococcus intestini RyC-MR95	GCF_000230275.1	*Haemophilus influenzae Rd KW20*	GCF_000027305.1
Acidaminococcus sp. AM33-14BH	GCF_003603525.1	*Haemophilus parainfluenzae T3T1*	GCF_000210895.1
Acidipropionibacterium acidipropionici CGMCC 1.2230	GCF_001441165.1	*Haemophilus quentini MP1*	GCF_001702075.1
Acidipropionibacterium jensenii DSM 20535	GCF_000425285.1	*Haemophilus sputorum CCUG 13788*	GCF_000238795.1
Acinetobacter baumannii AB030	GCF_000746645.1	*Hafnia alvei FB1*	GCF_000597785.2
Acinetobacter calcoaceticus CA16	GCF_002055515.1	*Helicobacter bilis AAQJH*	GCF_001999985.1
Acinetobacter haemolyticus HW-2A	GCF_003323815.1	*Helicobacter bizzozeronii CIII-1*	GCF_000237285.1
Acinetobacter johnsonii XBB1	GCF_001484935.1	*Helicobacter canadensis MIT 98-5491*	GCF_000162575.1
Acinetobacter junii 65	GCF_001941805.1	*Helicobacter cinaedi CCUG 18818 = ATCC BAA-847*	GCF_000349975.1
Acinetobacter lwoffii WJ10621	GCF_000219275.1	*Helicobacter hepaticus ATCC 51449*	GCF_000007905.1
Acinetobacter pittii PHEA-2	GCF_000191145.1	*Helicobacter pullorum MIT 98-5489*	GCF_000155495.1
Acinetobacter radioresistens DSM 6976	GCF_000368905.1	*Helicobacter pylori 26695*	GCF_000008525.1
Actinobacillus pleuropneumoniae serovar 8	GCF_001460855.1	*Helicobacter winghamensis ATCC BAA-430*	GCF_000158455.1
Actinomyces canis OH770	GCF_003858455.1	*Hespellia stercorisuis DSM 15480*	GCF_900142165.1

Actinomyces cardiffensis F0333	GCF_000364865.1	*Holdemanella biformis DSM 3989*	GCF_000156655.1
Actinomyces georgiae DSM 6843	GCF_000429245.1	*Holdemania filiformis DSM 12042*	GCF_000157995.1
Actinomyces graevenitzii C83	GCF_000239695.1	*Holdemania massiliensis AP2*	GCF_000327285.1
Actinomyces naeslundii NCTC 10301	GCF_001956585.1	*Hungateiclostridium clariflavum DSM 19732*	GCF_000237085.1
Actinomyces odontolyticus ATCC 17982	GCF_000154225.1	*Hungateiclostridium thermocellum ATCC 27405*	GCF_000015865.1
Actinomyces oris T14V	GCF_001553935.1	*Hungatella hathewayi WAL-18680*	GCF_000235505.1
Actinomyces radingae DSM 9169	GCF_900106055.1	*Hydrogenibacillus schlegelii MA 48*	GCF_001653195.1
Actinomyces sp. 2129	GCF_003606385.1	*Hydrogenoanaerobacterium saccharovorans CGMCC 1.5070*	GCF_900110045.1
Actinomyces turicensis ACS-279-V-Col4	GCF_000296505.1	*Hymenobacter rigui KCTC 12533*	GCF_003944715.1
Actinomyces urogenitalis DSM 15434	GCF_000159035.1	*Intestinimonas butyriciproducens AF211*	GCF_001454945.1
Actinomyces viscosus NCTC10951	GCF_900637975.1	*Janibacter limosus NBRC 16128*	GCF_001570985.1
Adlercreutzia equolifaciens DSM 19450	GCF_000478885.1	*Kallipyga massiliensis ph2*	GCF_000311985.1
Aeribacillus pallidus 8m3	GCF_001629795.1	*Kandleria vitulina DSM 20405*	GCF_000702065.1
Aerococcus viridans CCUG4311	GCF_001543285.1	*Klebsiella aerogenes KCTC 2190*	GCF_000215745.1
Aeromicrobium massiliense JC14	GCF_000312105.1	*Klebsiella oxytoca CAV1374*	GCF_001022195.1
Aeromonas allosaccharophila CECT 4199	GCF_000819685.1	*Klebsiella pneumoniae subsp. pneumoniae HS11286*	GCF_000240185.1
Aeromonas bestiarum CECT 4227	GCF_000819745.1	*Klebsiella sp. LY*	GCF_004010915.1
Aeromonas caviae GSH8M-1	GCF_003925855.1	*Klebsiella variicola At-22*	GCF_000025465.1
Aeromonas enteropelogenes CECT 4255T	GCF_000820205.1	*Kluyvera ascorbata ATCC 33433*	GCF_000735365.1
Aeromonas hydrophila subsp. hydrophila ATCC 7966	GCF_000014805.1	*Kocuria kristinae NBRC 15354*	GCF_001570865.1
Aeromonas jandaei IMET J	GCF_002925765.1	*Kocuria marina*	GCF_900169805.1
Aeromonas media WS	GCF_000287215.2	*Kocuria palustris MU14/1*	GCF_001275345.1
Aeromonas tecta CECT 7082	GCF_000820185.1	*Kocuria rhizophila DC2201*	GCF_000010285.1
Aeromonas veronii B565	GCF_000204115.1	*Kocuria rosea NCTC7512*	GCF_900638085.1
Agathobaculum desmolans ATCC 43058	GCF_000701665.1	*Krasilnikoviella flava DSM 21481*	GCF_900167525.1
Aggregatibacter aphrophilus ATCC 33389	GCF_900636915.1	*Kurthia gibsonii B83*	GCF_003660425.1
Aggregatibacter segnis ATCC 33393	GCF_900476035.1	*Kurthia massiliensis JC30T*	GCF_000285555.1
Agrococcus jejuensis DSM 22002	GCF_900099705.1	*Kurthia senegalensis JC8E*	GCF_000285595.1
Akkermansia muciniphila ATCC BAA-835	GCF_000020225.1	*Kurthia sp. 11kri321*	GCF_001534765.1
Alistipes finegoldii DSM 17242	GCF_000265365.1	*Kytococcus schroeteri UMB1298*	GCF_002847825.1
Alistipes indistinctus YIT 12060	GCF_000231275.1	*Kytococcus sedentarius DSM 20547*	GCF_000023925.1
Alistipes obesi	GCF_000311925.1	*Lachnoanaerobaculum saburreum DSM 3986*	GCF_000185385.1
Alistipes onderdonkii WAL 8169 = DSM 19147	GCF_000374505.1	*Lachnoanaerobaculum umeaense DSM 23576 = CCUG 58757*	GCF_003589745.1
Alistipes putredinis DSM 17216	GCF_000154465.1	*Lachnobacterium bovis DSM 14045*	GCF_900107245.1
Alistipes senegalensis JC50	GCF_000312145.1	*Lachnospira pectinoschiza M83*	GCF_900103815.1
Alistipes shahii WAL 8301	GCF_000210575.1	*Lachnospiraceae bacterium KH1P17*	GCF_900168235.1
Alistipes sp. Marseille-P5997	GCF_900604385.1	*Lactobacillus acidophilus NCFM*	GCF_000011985.1
Alistipes timonensis JC136	GCF_900107675.1	*Lactobacillus alimentarius DSM 20249*	GCF_001434745.1
Allisonella histaminiformans DSM 15230	GCF_900103425.1	*Lactobacillus amylolyticus L6*	GCF_002075105.1
Alloprevotella tannerae ATCC 51259	GCF_000159995.1	*Lactobacillus amylovorus 30SC*	GCF_000191545.1
Anaerobiospirillum succiniciproducens DSM 6400	GCF_000482845.1	*Lactobacillus animalis KCTC 3501 = DSM 20602*	GCF_001434535.1
Anaerobiospirillum thomasii NCTC12467	GCF_900445255.1	*Lactobacillus antri DSM 16041*	GCF_000160835.1
Anaerococcus hydrogenalis UMB0204	GCF_002871895.1	*Lactobacillus brevis ATCC 367*	GCF_000014465.1
Anaerococcus obesiensis ph10	GCF_000311745.1	*Lactobacillus buchneri CD034*	GCF_000298115.2
Anaerococcus octavius UMB0119	GCF_002847745.1	*Lactobacillus casei LcY*	GCF_000388095.2
Anaerococcus prevotii DSM 20548	GCF_000024105.1	*Lactobacillus coleohominis 101-4-CHN*	GCF_000161935.1
Anaerococcus senegalensis JC48	GCF_000321005.1	*Lactobacillus coryniformis subsp. coryniformis DSM 20001*	GCF_001433765.1
Anaerococcus tetradius ATCC 35098	GCF_000159095.1	*Lactobacillus crispatus ST1*	GCF_000091765.1
Anaerococcus vaginalis PH9	GCF_000307225.1	*Lactobacillus curvatus MRS6*	GCF_002224425.1
Anaerocolumna aminovalerica DSM 1283	GCF_900115365.1	*Lactobacillus delbrueckii subsp. bulgaricus ATCC 11842*	GCF_000056065.1
Anaerofustis stercorihominis DSM 17244	GCF_000154825.1	*Lactobacillus fermentum IFO 3956*	GCF_000010145.1
Anaerorhabdus furcosa ATCC 25662	GCF_900167375.1	*Lactobacillus fructivorans ATCC 27394*	GCF_001437175.1
Anaerostipes caccae DSM 14662	GCF_000154305.1	*Lactobacillus gasseri ATCC 33323 = JCM 1131*	GCF_000014425.1
Anaerostipes hadrus BPB5	GCF_001998765.1	*Lactobacillus gastricus DSM 16045*	GCF_001434365.1
Anaerostipes sp. AF04-45	GCF_003461725.1	*Lactobacillus helveticus CAUH18*	GCF_001308285.1
Anaerotignum lactatifermentans DSM 14214	GCF_900142265.1	*Lactobacillus hilgardii DSM 20176 = ATCC 8290*	GCF_000159315.1
Anaerotruncus colihominis DSM 17241	GCF_000154565.1	*Lactobacillus iners DSM 13335*	GCF_000160875.1
Aneurinibacillus aneurinilyticus ATCC 12856	GCF_000466385.1	*Lactobacillus jensenii SNUV360*	GCF_001936235.1
Aneurinibacillus migulanus DSM 2895	GCF_001274715.1	*Lactobacillus johnsonii DPC 6026*	GCF_000204985.1
Arcanobacterium haemolyticum DSM 20595	GCF_000092365.1	*Lactobacillus kalixensis DSM 16043*	GCF_001434335.1

Arcobacter butzleri RM4018	GCF_000014025.1	*Lactobacillus mucosae LM1*	GCF_000248095.2
Arcobacter cryaerophilus L406	GCF_001572875.1	*Lactobacillus oris F0423*	GCF_000221505.1
Arthrobacter castelli DSM 16402	GCF_000430705.1	*Lactobacillus parabuchneri FAM21731*	GCF_001922025.1
Asaccharobacter celatus AP38TSA	GCF_003340305.1	*Lactobacillus paracasei ATCC 334*	GCF_000014525.1
Asaccharospora irregularis DSM 2635	GCF_900129815.1	*Lactobacillus pentosus KCA1*	GCF_000271445.1
Atlantibacter hermannii NBRC 105704	GCF_000248015.1	*Lactobacillus plantarum WCFS1*	GCF_000203855.3
Atopobium minutum DSM 20586	GCF_001437015.1	*Lactobacillus reuteri DSM 20016*	GCF_000016825.1
Atopobium parvulum DSM 20469	GCF_000024225.1	*Lactobacillus rhamnosus GG*	GCF_000026505.1
Atopobium rimae ATCC 49626	GCF_000174015.1	*Lactobacillus ruminis ATCC 27782*	GCF_000224985.1
Atopobium vaginae DSM 15829	GCF_000159235.2	*Lactobacillus sakei FAM18311*	GCF_002224565.1
Bacillus altitudinis SGAir0031	GCF_002443015.2	*Lactobacillus salivarius UCC118*	GCF_000008925.1
Bacillus amyloliquefaciens DSM 7	GCF_000196735.1	*Lactobacillus saniviri JCM 17471 = DSM 24301*	GCF_001311785.1
Bacillus atrophaeus SRCM101359	GCF_002173495.1	*Lactobacillus senioris DSM 24302 = JCM 17472*	GCF_001436555.1
Bacillus badius SGD-V-25	GCF_001630115.1	*Lactobacillus sharpeae JCM 1186 = DSM 20505*	GCF_001436225.1
Bacillus cereus ATCC 14579	GCF_000007825.1	*Lactobacillus sp. wkB8*	GCF_000761135.1
Bacillus circulans NBRC 13626	GCF_001591585.1	*Lactobacillus ultunensis DSM 16047*	GCF_000159415.1
Bacillus clausii KSM-K16	GCF_000009825.1	*Lactobacillus vaginalis DSM 5837 = ATCC 49540*	GCF_000159435.1
Bacillus coagulans DSM 1 = ATCC 7050	GCF_000832905.1	*Lactococcus garvieae ATCC 49156*	GCF_000269925.1
Bacillus endophyticus DSM 13796	GCF_900115845.1	*Lactococcus lactis subsp. lactis Il1403*	GCF_000006865.1
Bacillus firmus 14_TX	GCF_003315495.1	*Lactococcus plantarum DSM 20686*	GCF_002441715.1
Bacillus flexus KLBMP 4941	GCF_002024265.1	*Lactococcus raffinolactis WiKim0068*	GCF_002310475.1
Bacillus fordii DSM 16014 = CIP 108821	GCF_000374565.1	*Lactonifactor longoviformis DSM 17459*	GCF_900129135.1
Bacillus halodurans C-125	GCF_000011145.1	*Leminorella grimontii ATCC 33999 = DSM 5078*	GCF_000439085.1
Bacillus halotolerans ZB201702	GCF_004006435.1	*Leminorella richardii NCTC12151*	GCF_900478135.1
Bacillus infantis NRRL B-14911	GCF_000473245.1	*Leptotrichia buccalis C-1013-b*	GCF_000023905.1
Bacillus licheniformis DSM 13 = ATCC 14580	GCF_000011645.1	*Leptotrichia hofstadii F0254*	GCF_000162955.1
Bacillus marisflavi JCM 11544	GCF_001274775.1	*Leuconostoc gelidum subsp. gasicomitatum LMG 18811*	GCF_000196855.1
Bacillus massilioanorexius AP8	GCF_000321185.1	*Leuconostoc mesenteroides subsp. mesenteroides ATCC 8293*	GCF_000014445.1
Bacillus massiliosenegalensis JC6	GCF_000311725.1	*Listeria grayi DSM 20601*	GCF_000148995.1
Bacillus megaterium NBRC 15308 = ATCC 14581	GCF_000832985.1	*Listeria innocua FSL J1-023*	GCF_000183905.1
Bacillus mojavensis RO-H-1 = KCTC 3706	GCF_000507105.1	*Listeria monocytogenes EGD-e*	GCF_000196035.1
Bacillus mycoides ATCC 6462	GCF_000832605.1	*Lysinibacillus fusiformis RB-21*	GCF_000724775.3
Bacillus nealsonii AAU1	GCF_000401235.1	*Lysinibacillus massiliensis 4400831*	GCF_000772965.1
Bacillus niacini NBRC 15566	GCF_001591505.1	*Lysinibacillus sphaericus LMG 22257*	GCF_001753205.1
Bacillus pseudofirmus OF4	GCF_000005825.2	*Lysobacter soli KCTC 22011*	GCF_003382285.1
Bacillus pumilus SH-B9	GCF_001578205.1	*Marvinbryantia formatexigens DSM 14469*	GCF_000173815.1
Bacillus simplex SH-B26	GCF_001578185.1	*Megamonas funiformis YIT 11815*	GCF_000245775.1
Bacillus smithii DSM 4216	GCF_001050115.1	*Megamonas hypermegale DSM 1672*	GCF_000423385.1
Bacillus sonorensis SRCM101395	GCF_002202015.1	*Megasphaera elsdenii 14-14*	GCF_001304715.1
Bacillus sp. FJAT-42376	GCF_003816055.1	*Megasphaera micronuciformis F0359*	GCF_000165735.1
Bacillus subtilis subsp. subtilis str. 168	GCF_000009045.1	*Metakosakonia massiliensis JC163*	GCF_000321045.1
Bacillus thermoamylovorans 1A1	GCF_000751775.1	*Microbacterium aurum KACC 15219*	GCF_001974985.1
Bacillus thuringiensis YBT-1518	GCF_000497525.1	*Microbacterium chocolatum SIT 101*	GCF_001652465.1
Bacillus timonensis MM10403188	GCF_000285535.1	*Microbacterium foliorum DSM 12966*	GCF_000956415.1
Bacillus vallismortis Bac111	GCF_003667885.1	*Microbacterium gubbeenense DSM 15944*	GCF_000422745.1
Bacillus velezensis AP183	GCF_000875875.2	*Microbacterium hydrocarbonoxydans SA35*	GCF_000956475.1
Bacteroides caccae ATCC 43185	GCF_002222615.2	*Microbacterium oleivorans NBRC 103075*	GCF_001552475.1
Bacteroides cellulosilyticus WH2	GCF_001318345.1	*Microbacterium paraoxydans DSM 15019*	GCF_900105335.1
Bacteroides clarus YIT 12056	GCF_000195615.1	*Microbacterium phyllosphaerae A1.1*	GCF_003614915.1
Bacteroides coprocola DSM 17136	GCF_000154845.1	*Micrococcus luteus NCTC 2665*	GCF_000023205.1
Bacteroides coprophilus DSM 18228 = JCM 13818	GCF_000157915.1	*Micrococcus lylae NBRC 15355*	GCF_001570885.1
Bacteroides dorei CL03T12C01	GCF_001640865.1	*Micromonospora aurantiaca ATCC 27029*	GCF_000145235.1
Bacteroides eggerthii DSM 20697	GCF_000155815.1	*Mitsuokella jalaludinii 2789STDY5608828*	GCF_001404615.1
Bacteroides faecis MAJ27	GCF_900106755.1	*Mitsuokella multacida DSM 20544*	GCF_000155955.1
Bacteroides finegoldii DSM 17565	GCF_000156195.1	*Mobiluncus curtisii ATCC 43063*	GCF_000196535.1
Bacteroides fluxus YIT 12057	GCF_000195635.1	*Mobiluncus mulieris ATCC 35243*	GCF_000160615.1
Bacteroides fragilis YCH46	GCF_000009925.1	*Moellerella wisconsensis ATCC 35017*	GCF_001294465.1
Bacteroides graminisolvens DSM 19988 = JCM 15093	GCF_000428125.1	*Mogibacterium diversum CCUG 47132*	GCF_002998925.1
Bacteroides intestinalis AF31-23	GCF_003475505.1	*Mogibacterium timidum ATCC 33093*	GCF_000525775.1
Bacteroides massiliensis B84634	GCF_000382445.1	*Moraxella catarrhalis BBH18*	GCF_000092265.1

Bacteroides nordii CL02T12C05	GCF_000273175.1	*Moraxella osloensis CCUG 350*	GCF_001553955.1
Bacteroides oleiciplenus YIT 12058	GCF_000315485.1	*Morganella morganii subsp. morganii KT*	GCF_000286435.2
Bacteroides ovatus ATCC 8483	GCF_001314995.1	*Mycobacterium avium subsp. paratuberculosis K-10*	GCF_000007865.1
Bacteroides pectinophilus ATCC 43243	GCF_000155855.1	*Mycobacterium florentinum DSM 44852*	GCF_002101635.1
Bacteroides plebeius DSM 17135	GCF_000187895.1	*Mycobacterium tuberculosis H37Rv*	GCF_000195955.2
Bacteroides pyogenes JCM 10003	GCF_000511775.1	*Mycobacteroides abscessus*	GCF_000069185.1
Bacteroides salanitronis DSM 18170	GCF_000190575.1	*Mycolicibacterium fortuitum CT6*	GCF_001307545.1
Bacteroides salyersiae WAL 10018	GCF_000381365.1	*Mycoplasma hominis ATCC 23114*	GCF_000085865.1
Bacteroides sp. 4_1_36 TM07-1	GCF_003466325.1	*Mycoplasma pneumoniae M129*	GCF_000027345.1
Bacteroides stercoris ATCC 43183	GCF_000154525.1	*Neisseria cinerea NCTC10294*	GCF_900475315.1
Bacteroides thetaiotaomicron VPI-5482	GCF_000011065.1	*Neisseria elongata subsp. glycolytica ATCC 29315*	GCF_000818035.1
Bacteroides timonensis	GCF_000513195.1	*Neisseria flavescens SK114*	GCF_000175275.1
Bacteroides uniformis ATCC 8492	GCF_000154205.1	*Neisseria macacae ATCC 33926*	GCF_000220865.1
Bacteroides vulgatus ATCC 8482	GCF_000012825.1	*Neisseria mucosa C102*	GCF_000186165.1
Bacteroides xylanisolvens SD CC 2a	GCF_000577955.1	*Neisseria perflava UMB0023*	GCF_002863305.1
Barnesiella intestinihominis YIT 11860	GCF_000296465.1	*Neisseria sicca FDAARGOS_260*	GCF_002073715.2
Bhargavaea beijingensis CGMCC 1.6762	GCF_900101985.1	*Neisseria subflava M18660*	GCF_003351665.1
Bifidobacterium adolescentis ATCC 15703	GCF_000010425.1	*Nevskia ramosa DSM 11499*	GCF_000420645.1
Bifidobacterium angulatum DSM 20098 = JCM 7096	GCF_001025155.1	*Oceanobacillus caeni HM6*	GCF_001298135.1
Bifidobacterium animalis subsp. lactis DSM 10140	GCF_000022965.1	*Oceanobacillus massiliensis str. N'diop*	GCF_000285495.1
Bifidobacterium bifidum PRL2010	GCF_000165905.1	*Odoribacter laneus YIT 12061*	GCF_000243215.1
Bifidobacterium boum DSM 20432	GCF_000687615.1	*Odoribacter splanchnicus DSM 20712*	GCF_000190535.1
Bifidobacterium breve DSM 20213 = JCM 1192	GCF_001025175.1	*Olsenella profusa F0195*	GCF_000468755.1
Bifidobacterium catenulatum DSM 16992	GCF_001025195.1	*Olsenella uli DSM 7084*	GCF_000143845.1
Bifidobacterium coryneforme LMG18911	GCF_000737865.1	*Oribacterium sinus F0268*	GCF_000160635.1
Bifidobacterium dentium JCM 1195 = DSM 20436	GCF_001042595.1	*Oscillibacter valericigenes Sjm18-20*	GCF_000283575.1
Bifidobacterium gallicum DSM 20093 = LMG 11596	GCF_000741205.1	*Paenibacillus alvei DSM 29*	GCF_000293805.1
Bifidobacterium kashiwanohense PV20-2	GCF_000800455.1	*Paenibacillus antibioticophila GD11*	GCF_000455265.1
Bifidobacterium longum NCC2705	GCF_000007525.1	*Paenibacillus barcinonensis CECT 7022*	GCF_003217495.1
Bifidobacterium mongoliense DSM 21395	GCF_000741285.1	*Paenibacillus barengoltzii G22*	GCF_000403375.2
Bifidobacterium pseudocatenulatum DSM 20438	GCF_001025215.1	*Paenibacillus daejeonensis DSM 15491*	GCF_000378385.1
Bifidobacterium pseudolongum PV8-2	GCF_000800475.2	*Paenibacillus durus DSM 1735*	GCF_000756615.1
Bifidobacterium ruminantium LMG 21811	GCF_000741365.1	*Paenibacillus glucanolyticus 5162*	GCF_001632305.1
Bifidobacterium scardovii JCM 12489 = DSM 13734	GCF_001042635.1	*Paenibacillus graminis DSM 15220*	GCF_000758705.1
Bifidobacterium sp. AGR2158	GCF_000424225.1	*Paenibacillus illinoisensis NBRC 15959*	GCF_004000925.1
Bifidobacterium stercoris JCM 15918	GCF_000771705.1	*Paenibacillus lactis 154*	GCF_000230915.1
Bifidobacterium thermacidophilum subsp. Thermacidophilum LMG 21395	GCF_000741455.1	*Paenibacillus lautus E7593-69*	GCF_003590055.1
Bifidobacterium thermophilum RBL67	GCF_000347695.1	*Paenibacillus rhizosphaerae FSL R5-0378*	GCF_001956185.1
Bilophila sp. 4_1_30	GCF_000224655.1	*Paenibacillus senegalensis JC66*	GCF_000285515.1
Bilophila wadsworthia ATCC 49260	GCF_000701705.1	*Paenibacillus sp. RUD330*	GCF_002243345.1
Blautia coccoides YL58	GCF_002221555.2	*Paenibacillus thiaminolyticus BO5*	GCF_003591545.1
Blautia hansenii DSM 20583	GCF_000156675.1	*Paeniclostridium sordellii CBA7122*	GCF_001950115.1
Blautia hydrogenotrophica DSM 10507	GCF_000157975.1	*Paenisporosarcina sp. K2R23-3*	GCF_003595195.1
Blautia massiliensis GD9	GCF_001487165.1	*Pantoea agglomerans C410P1*	GCF_001709315.1
Blautia obeum ATCC 29174	GCF_000153905.1	*Papillibacter cinnamivorans DSM 12816*	GCF_900176335.1
Blautia producta ATCC 27340 = DSM 2950	GCF_000373885.1	*Parabacteroides distasonis ATCC 8503*	GCF_000012845.1
Blautia wexlerae DSM 19850	GCF_000484655.1	*Parabacteroides goldsteinii DSM 19448 = WAL 12034*	GCF_000969835.1
Brachybacterium paraconglomeratum LC44	GCF_000233655.1	*Parabacteroides gordonii DSM 23371*	GCF_000428565.1
Brachyspira pilosicoli P43/6/78	GCF_000325665.1	*Parabacteroides johnsonii CL02T12C29*	GCF_000307375.1
Brevibacillus agri 5-2	GCF_000612125.1	*Parabacteroides merdae ATCC 43184*	GCF_000154105.1
Brevibacillus borstelensis AK1	GCF_000353565.1	*Parabacteroides sp. CT06*	GCF_002257605.1
Brevibacillus brevis NBRC 100599	GCF_000010165.1	*Paraclostridium bifermentans ATCC 638*	GCF_000452245.2
Brevibacillus massiliensis	GCF_000311785.1	*Paraeggerthella hongkongensis RC2/2 A*	GCF_003340345.1
Brevibacterium casei S18	GCF_000314575.1	*Paraprevotella clara YIT 11840*	GCF_000233955.1
Brevibacterium epidermidis EZ-K02	GCF_002573745.1	*Paraprevotella xylaniphila YIT 11841*	GCF_000205165.1
Brevibacterium iodinum ATCC 49514	GCF_900169265.1	*Parasporobacterium paucivorans DSM 15970*	GCF_900141895.1
Brevibacterium linens BS258	GCF_001606005.1	*Parasutterella excrementihominis YIT 11859*	GCF_000205025.1
Brevibacterium ravenspurgense 5401308 = CCUG 53855	GCF_000285915.1	*Parvimonas micra KCOM 1535; ChDC B708*	GCF_000800295.1
Brevibacterium senegalense	GCF_000285835.1	*Parvimonas sp. KA00067*	GCF_001553085.1

Brochothrix thermosphacta DSM 20171 = FSL F6-1036	GCF_000620985.1	*Pediococcus acidilactici ZPA017*	GCF_001767275.1
Bulleidia extructa W1219	GCF_000177375.1	*Pediococcus damnosus TMW 2.1535*	GCF_001611135.1
Burkholderiales bacterium JOSHI_001	GCF_000244995.1	*Pediococcus pentosaceus ATCC 25745*	GCF_000014505.1
Butyricicoccus pullicaecorum 1.2	GCF_000398925.1	*Peptococcus niger DSM 20475*	GCF_900101835.1
Butyricimonas synergistica DSM 23225	GCF_000379665.1	*Peptoniphilus asaccharolyticus DSM 20463*	GCF_900176115.1
Butyricimonas virosa DSM 23226	GCF_000519105.1	*Peptoniphilus duerdenii ATCC BAA-1640*	GCF_000146345.1
Butyrivibrio crossotus DSM 2876	GCF_000156015.1	*Peptoniphilus grossensis ph5*	GCF_000311825.1
Butyrivibrio fibrisolvens DSM 3071	GCF_900129945.1	*Peptoniphilus harei ACS-146-V-Sch2b*	GCF_000183565.1
Campylobacter coli aerotolerant OR12	GCF_002024185.1	*Peptoniphilus indolicus ATCC 29427*	GCF_000227315.1
Campylobacter concisus ATCC 33237	GCF_001298465.1	*Peptoniphilus lacrimalis DSM 7455*	GCF_000378725.1
Campylobacter curvus 525.92	GCF_000017465.2	*Peptoniphilus senegalensis JC140*	GCF_000321025.1
Campylobacter fetus subsp. testudinum 03-427	GCF_000495505.1	*Peptoniphilus sp. ING2-D1G*	GCF_000952975.1
Campylobacter gracilis ATCC 33236	GCF_001190745.1	*Peptoniphilus timonensis JC401*	GCF_000312025.1
Campylobacter hominis ATCC BAA-381	GCF_000017585.1	*Peptostreptococcus anaerobius VPI 4330 = DSM 2949*	GCF_000381525.1
Campylobacter hyointestinalis subsp. Lawsonii LMG 15993	GCF_001643975.1	*Peptostreptococcus stomatis DSM 17678*	GCF_000147675.1
Campylobacter jejuni subsp. jejuni NCTC 11168	GCF_000009085.1	*Phascolarctobacterium faecium JCM 30894*	GCF_003945365.1
Campylobacter lari RM2100	GCF_000019205.1	*Phascolarctobacterium succinatutens YIT 12067*	GCF_000188175.1
Campylobacter rectus RM3267	GCF_000174175.1	*Phyllobacterium myrsinacearum IAM 13584*	GCF_003182235.1
Campylobacter showae CSUNSWCD	GCF_000313615.1	*Phyllobacterium sp. UNC302MFCol5.2*	GCF_000686005.1
Campylobacter sp. 10_1_50	GCF_000238755.1	*Planococcus rifietoensis M8*	GCF_001465795.2
Campylobacter upsaliensis DSM 5365	GCF_000620965.1	*Plesiomonas shigelloides NCTC10360*	GCF_900087055.1
Campylobacter ureolyticus RIGS 9880	GCF_001190755.1	*Porphyromonas asaccharolytica DSM 20707*	GCF_000212375.1
Candidatus Blastococcus massiliensis AP3	GCF_000582785.1	*Porphyromonas endodontalis ATCC 35406*	GCF_000174815.1
Candidatus Dorea massiliensis AP6	GCF_001282705.1	*Porphyromonas gingivalis ATCC 33277*	GCF_000010505.1
Candidatus Soleaferrea massiliensis AP7	GCF_000820765.1	*Porphyromonas somerae KA00683*	GCF_001553215.1
Candidatus Stoquefichus massiliensis AP9	GCF_000455285.1	*Porphyromonas uenonis DSM 23387 = JCM 13868*	GCF_000482365.1
Capnocytophaga gingivalis ATCC 33624	GCF_000174755.1	*Prevotella albensis DSM 11370 = JCM 12258*	GCF_000426565.1
Capnocytophaga granulosa ATCC 51502	GCF_000411115.1	*Prevotella amnii DSM 23384 = JCM 14753*	GCF_000378745.1
Capnocytophaga ochracea DSM 7271	GCF_000023285.1	*Prevotella bivia DSM 20514*	GCF_000262545.1
Capnocytophaga sputigena NCTC11097	GCF_900638125.1	*Prevotella brevis ATCC 19188*	GCF_000621725.1
Cardiobacterium hominis ATCC 15826	GCF_000160655.1	*Prevotella bryantii B14*	GCF_000179055.1
Catabacter hongkongensis HKU16	GCF_000981035.1	*Prevotella buccae ATCC 33574*	GCF_000184945.1
Catenibacterium mitsuokai DSM 15897	GCF_000173795.1	*Prevotella conceptionensis 9403948*	GCF_000312305.1
Catonella morbi ATCC 51271	GCF_000160035.2	*Prevotella copri DSM 18205*	GCF_000157935.1
Cedecea davisae DSM 4568	GCF_000412335.2	*Prevotella corporis DSM 18810 = JCM 8529*	GCF_000430525.1
Cellulomonas massiliensis JC225	GCF_000312005.1	*Prevotella denticola F0289*	GCF_000193395.1
Cellulosilyticum lentocellum DSM 5427	GCF_000178835.2	*Prevotella disiens JCM 6334 = ATCC 29426*	GCF_000613345.1
Cellulosimicrobium cellulans PSBB019	GCF_002162335.1	*Prevotella enoeca F0113*	GCF_001444445.1
Cetobacterium somerae ATCC BAA-474	GCF_000479045.1	*Prevotella intermedia ATCC 25611 = DSM 20706*	GCF_001953955.1
Christensenella minuta DSM 22607	GCF_001652705.1	*Prevotella loescheii DSM 19665 = JCM 12249 = ATCC 15930*	GCF_000378085.1
Chryseobacterium hominis DSM 19326	GCF_900108525.1	*Prevotella marshii DSM 16973 = JCM 13450*	GCF_000146675.1
Citrobacter amalonaticus Y19	GCF_000981805.1	*Prevotella melaninogenica ATCC 25845*	GCF_000144405.1
Citrobacter braakii FDAARGOS_290	GCF_002208845.2	*Prevotella nanceiensis DSM 19126 = JCM 15639*	GCF_000379965.1
Citrobacter farmeri AUSMDU00008141	GCF_002249995.1	*Prevotella nigrescens ATCC 33563*	GCF_000220235.1
Citrobacter freundii CFNIH1	GCF_000648515.1	*Prevotella oralis ATCC 33269*	GCF_000185145.2
Citrobacter gillenii C3	GCF_003429605.1	*Prevotella oulorum JCM 14966*	GCF_000613785.1
Citrobacter koseri ATCC BAA-895	GCF_000018045.1	*Prevotella pallens ATCC 700821*	GCF_000220255.1
Citrobacter sedlakii NBRC 105722	GCF_000759835.1	*Prevotella ruminicola 23*	GCF_000025925.1
Citrobacter sp. 92	GCF_900079795.1	*Prevotella salivae DSM 15606*	GCF_000185845.1
Citrobacter werkmanii UMH18	GCF_003665555.1	*Prevotella shahii DSM 15611 = JCM 12083*	GCF_000613445.1
Citrobacter youngae NCTC13709	GCF_900638065.1	*Prevotella stercorea DSM 18206*	GCF_000235885.1
Cloacibacillus evryensis DSM 19522	GCF_000585335.1	*Prevotella timonensis 4401737 = DSM 22865 = JCM 15640*	GCF_000455445.1
Cloacibacterium normanense DSM 15886	GCF_900104195.1	*Prevotella veroralis DSM 19559 = JCM 6290*	GCF_000377625.1
Clostridiales bacterium WTE2008	GCF_900176495.1	*Propionibacterium freudenreichii subsp. Freudenreichii DSM 20271*	GCF_000940845.1
Clostridioides difficile 630	GCF_000009205.2	*Propionibacterium sp. oral taxon 193*	GCF_001717565.1
Clostridium acetobutylicum ATCC 824	GCF_000008765.1	*Proteus mirabilis HI4320*	GCF_000069965.1
Clostridium aldenense AM40-2AC	GCF_003467385.1	*Proteus penneri ATCC 33519*	GCF_003144535.1
Clostridium asparagiforme DSM 15981	GCF_000158075.1	*Proteus vulgaris FDAARGOS_556*	GCF_003812525.1
Clostridium baratii str. Sullivan	GCF_000789395.1	*Providencia alcalifaciens Dmel2*	GCF_000314875.2

Clostridium beijerinckii NCIMB 14988	GCF_000833105.2	*Providencia rettgeri Dmel1*	GCF_000314835.2
Clostridium bolteae 90A9	GCF_000371705.1	*Providencia rustigianii NCTC8113*	GCF_900637755.1
Clostridium botulinum A str. Hall	GCF_000017045.1	*Providencia stuartii MRSN 2154*	GCF_000259175.1
Clostridium butyricum KNU-L09	GCF_001456065.2	*Pseudoclavibacter sp. RFBG4*	GCF_002931515.1
Clostridium cadaveris AGR2141	GCF_000424205.1	*Pseudoflavonifractor capillosus ATCC 29799*	GCF_000169255.2
Clostridium celatum DSM 1785	GCF_000320405.1	*Pseudoglutamicibacter albus DNF00011*	GCF_000758985.1
Clostridium chauvoei JF4335	GCF_900168365.1	*Pseudomonas aeruginosa PAO1*	GCF_000006765.1
Clostridium citroniae WAL-17108	GCF_000233455.1	*Pseudomonas alcaliphila JAB1*	GCF_001941865.1
Clostridium clostridioforme ATCC 25537	GCF_900113155.1	*Pseudomonas fluorescens F113*	GCF_000237065.1
Clostridium cochlearium NLAE-zl-C224	GCF_900103025.1	*Pseudomonas monteilii USDA-ARS-USMARC-56711*	GCF_001534745.1
Clostridium cocleatum DSM 1551	GCF_900102365.1	*Pseudomonas nitroreducens NBRC 12694*	GCF_002091755.1
Clostridium disporicum 2789STDY5834856	GCF_001405015.1	*Pseudomonas oleovorans MOIL14HWK12*	GCF_000510765.1
Clostridium fallax DSM 2631	GCF_900129365.1	*Pseudomonas putida KT2440*	GCF_000007565.2
Clostridium felsineum DSM 794	GCF_002006355.1	*Pseudomonas sp. 09C 129*	GCF_002843625.1
Clostridium glycyrrhizinilyticum JCM 13369	GCF_001311035.1	*Pseudomonas stutzeri CGMCC 1.1803*	GCF_000219605.1
Clostridium hiranonis DSM 13275	GCF_000156055.1	*Pseudopropionibacterium propionicum F0230a*	GCF_000277715.1
Clostridium hylemonae DSM 15053	GCF_000156515.1	*Pseudoramibacter alactolyticus ATCC 23263*	GCF_000185505.1
Clostridium indolis DSM 755	GCF_000526995.1	*Pseudoxanthomonas mexicana CCH9-G4*	GCF_001556105.1
Clostridium innocuum 2959	GCF_000371425.1	*Pyramidobacter piscolens W5455*	GCF_000177335.1
Clostridium lavalense NLAE-zl-G277	GCF_900102595.1	*Raoultella planticola FDAARGOS_430*	GCF_003699975.1
Clostridium leptum DSM 753	GCF_000154345.1	*Raoultella terrigena BIGb0267*	GCF_003752645.1
Clostridium methoxybenzovorans SR3	GCF_000421505.1	*Rhodococcus erythropolis CCM2595*	GCF_000454045.1
Clostridium methylpentosum DSM 5476	GCF_000158655.1	*Rhodococcus hoagii 103S*	GCF_000196695.1
Clostridium neonatale LCDC no.99-A-005	GCF_001458595.1	*Rhodococcus rhodochrous EP4*	GCF_003004765.2
Clostridium paraputrificum AGR2156	GCF_000424025.1	*Rhodococcus sp. PBTS 1*	GCF_001620025.1
Clostridium perfringens ATCC 13124	GCF_000013285.1	*Robinsoniella peoriensis WT*	GCF_000797495.1
Clostridium populeti 743A	GCF_900112775.1	*Romboutsia lituseburensis DSM 797*	GCF_900103615.1
Clostridium putrefaciens NCTC9836	GCF_900461105.1	*Roseburia faecis 2789STDY5608863*	GCF_001405615.1
Clostridium saccharogumia DSM 17460	GCF_000686665.1	*Roseburia hominis A2-183*	GCF_000225345.1
Clostridium saccharolyticum WM1	GCF_000144625.1	*Roseburia intestinalis L1-82*	GCF_000156535.1
Clostridium saccharoperbutylacetonicum N1-4(HMT)	GCF_000340885.1	*Roseburia inulinivorans DSM 16841*	GCF_000174195.1
Clostridium sartagoforme AAU1	GCF_000401215.1	*Rothia aeria JCM 11412*	GCF_002355935.1
Clostridium scindens ATCC 35704	GCF_000154505.1	*Rothia dentocariosa ATCC 17931*	GCF_000164695.2
Clostridium senegalense JC122	GCF_000285575.1	*Rothia mucilaginosa DY-18*	GCF_000011025.1
Clostridium septicum DSM 7534	GCF_003606265.1	*Rudanella lutea DSM 19387*	GCF_000383955.1
Clostridium sp. DL-VIII	GCF_000230835.1	*Ruminiclostridium cellobioparum subsp. termitidis CT1112*	GCF_000350485.1
Clostridium sphenoides JCM 1415	GCF_900105615.1	*Ruminococcaceae bacterium KHP2*	GCF_900176545.1
Clostridium spiroforme DSM 1552	GCF_000154805.1	*Ruminococcus albus 7 = DSM 20455*	GCF_000179635.2
Clostridium sporogenes DSM 795	GCF_001020205.1	*Ruminococcus bromii YE282*	GCF_900101355.1
Clostridium sporosphaeroides DSM 1294	GCF_000383295.1	*Ruminococcus callidus ATCC 27760*	GCF_000468015.1
Clostridium symbiosum WAL-14163	GCF_000189595.1	*Ruminococcus champanellensis 18P13 = JCM 17042*	GCF_000210095.1
Clostridium tertium	GCF_900217175.1	*Ruminococcus faecis JCM 15917*	GCF_001312505.1
Clostridium tyrobutyricum KCTC 5387	GCF_001642655.1	*Ruminococcus flavefaciens MC2020*	GCF_000701945.1
Clostridium ventriculi 2789STDY5834858	GCF_001404895.1	*Ruminococcus gauvreauii DSM 19829*	GCF_000425525.1
Clostridium vincentii DSM 10228	GCF_002995745.1	*Ruminococcus gnavus AGR2154*	GCF_000526735.1
Clostridium viride DSM 6836	GCF_000620945.1	*Ruminococcus lactaris ATCC 29176*	GCF_000155205.1
Collinsella aerofaciens ATCC 25986	GCF_000169035.1	*Ruminococcus sp. YE71*	GCF_900119155.1
Collinsella intestinalis DSM 13280	GCF_000156175.1	*Ruminococcus torques ATCC 27756*	GCF_000153925.1
Collinsella stercoris DSM 13279	GCF_000156215.1	*Salmonella enterica subsp. enterica serovar Typhimurium str. LT2*	GCF_000006945.2
Collinsella tanakaei YIT 12063	GCF_000225705.1	*Scardovia inopinata JCM 12537*	GCF_001042695.1
Coprobacillus cateniformis OF01-6	GCF_003463235.1	*Schlesneria paludicola DSM 18645*	GCF_000255655.1
Coprobacillus sp. D7	GCF_000158555.2	*Selenomonas ruminantium subsp. lactilytica TAM6421*	GCF_000284095.1
Coprococcus catus AM28-39	GCF_003434235.1	*Selenomonas sp. oral taxon 149 str. 67H29BP*	GCF_000146365.1
Coprococcus comes ATCC 27758	GCF_000155875.1	*Senegalimassilia anaerobia JC110*	GCF_000236865.1
Coprococcus eutactus ATCC 27759	GCF_000154425.1	*Serratia ficaria NCTC12148*	GCF_900187015.1
Corynebacterium afermentans subsp. Afermentans LCDC880199	GCF_001639025.1	*Serratia fonticola DSM 4576*	GCF_001006005.1
Corynebacterium ammoniagenes DSM 20306	GCF_001941425.1	*Serratia liquefaciens ATCC 27592*	GCF_000422085.1
Corynebacterium amycolatum UMB0338	GCF_002861405.1	*Serratia marcescens subsp. marcescens Db11*	GCF_000513215.1
Corynebacterium appendicis CIP 107643	GCF_900156665.1	*Shewanella sp. M2*	GCF_003855155.1

Corynebacterium aurimucosum ATCC 700975	GCF_000022905.1	*Shigella boydii G1227*	GCF_003336935.1
Corynebacterium coyleae DSM 44184	GCF_900105505.1	*Shigella dysenteriae Sd197*	GCF_000012005.1
Corynebacterium durum F0235	GCF_000318135.1	*Shigella flexneri 2a str. 301*	GCF_000006925.2
Corynebacterium freneyi DNF00450	GCF_000758965.1	*Shigella sonnei KY 1629*	GCF_001261695.1
Corynebacterium glaucum DSM 30827	GCF_002287505.1	*Shigella sp. PAMC 28760*	GCF_001596115.1
Corynebacterium glucuronolyticum DSM 44120	GCF_900176155.1	*Silanimonas lenta DSM 16282*	GCF_000429065.1
Corynebacterium kroppenstedtii DSM 44385	GCF_000023145.1	*Slackia equolifaciens DSM 24851*	GCF_003725995.1
Corynebacterium matruchotii ATCC 14266	GCF_000175375.1	*Slackia exigua ATCC 700122*	GCF_000162875.1
Corynebacterium minutissimum NCTC10288	GCF_900478045.1	*Slackia isoflavoniconvertens OB21 GAM31*	GCF_003340315.1
Corynebacterium propinquum DSM 44285	GCF_000375525.1	*Slackia piriformis YIT 12062*	GCF_000296445.1
Corynebacterium pseudodiphtheriticum DSM 44287	GCF_000688415.1	*Sneathia amnii SN35*	GCF_000973085.1
Corynebacterium pseudogenitalium ATCC 33035	GCF_000156615.2	*Solobacterium moorei DSM 22971*	GCF_000425005.1
Corynebacterium simulans PES1	GCF_001586215.1	*Sphingobacterium multivorum NCTC11034*	GCF_900457115.1
Corynebacterium sp. HFH0082	GCF_000411235.1	*Sporosarcina koreensis Q1*	GCF_001531445.1
Corynebacterium striatum 216	GCF_002804085.1	*Staphylococcus arlettae CVD059*	GCF_000295715.1
Corynebacterium tuberculostearicum SK141	GCF_000175635.1	*Staphylococcus aureus subsp. aureus NCTC 8325*	GCF_000013425.1
Corynebacterium ulcerans FRC11	GCF_000767685.1	*Staphylococcus auricularis DSM 20609*	GCF_001500315.1
Corynebacterium ureicelerivorans IMMIB RIV-2301	GCF_000747315.1	*Staphylococcus capitis subsp. Capitis AYP1020*	GCF_001028645.1
Corynebacterium xerosis NBRC 16721	GCF_001552415.1	*Staphylococcus caprae JMUB898*	GCF_003966625.1
Cronobacter sakazakii ATCC 29544	GCF_000982825.1	*Staphylococcus cohnii subsp. Cohnii 532*	GCF_000972575.1
Cryptobacterium curtum DSM 15641	GCF_000023845.1	*Staphylococcus condimenti DSM 11674*	GCF_001618885.1
Curtobacterium flaccumfaciens UCD-AKU	GCF_000349565.1	*Staphylococcus epidermidis ATCC 12228*	GCF_000007645.1
Cutibacterium acnes KPA171202	GCF_000008345.1	*Staphylococcus equorum KS1039*	GCF_001432245.1
Cutibacterium avidum 44067	GCF_000367205.1	*Staphylococcus haemolyticus JCSC1435*	GCF_000009865.1
Cutibacterium granulosum DSM 20700	GCF_000463665.1	*Staphylococcus hominis subsp. hominis C80*	GCF_000183685.1
Deinococcus radiodurans R1	GCF_000008565.1	*Staphylococcus intermedius NCTC 11048*	GCF_002902385.1
Dermabacter hominis 1368	GCF_000775415.1	*Staphylococcus kloosii ATCC 43959*	GCF_003019255.1
Dermabacter sp. HFH0086	GCF_000413375.1	*Staphylococcus lugdunensis HKU09-01*	GCF_000025085.1
Dermacoccus nishinomiyaensis M25	GCF_000725405.1	*Staphylococcus pasteuri SP1*	GCF_000494875.1
Desulfitobacterium hafniense DCB-2	GCF_000021925.1	*Staphylococcus pettenkoferi FDAARGOS_288*	GCF_002208805.2
Desulfovibrio desulfuricans ND132	GCF_000189295.2	*Staphylococcus saccharolyticus NCTC 11807*	GCF_003970495.1
Desulfovibrio fairfieldensis CCUG 45958	GCF_001553605.1	*Staphylococcus saprophyticus subsp. saprophyticus ATCC 15305*	GCF_000010125.1
Desulfovibrio piger	GCF_900116045.1	*Staphylococcus schleiferi OT1-1*	GCF_004026205.1
Desulfovibrio sp. FW1012B	GCF_000177215.2	*Staphylococcus sciuri FDAARGOS_285*	GCF_002209165.2
Dialister invisus DSM 15470	GCF_000160055.1	*Staphylococcus simulans FDAARGOS_124*	GCF_001559115.2
Dialister micraerophilus DSM 19965	GCF_000194985.1	*Staphylococcus sp. AntiMn-1*	GCF_001663395.1
Dialister microaerophilus UPII 345-E	GCF_000183445.1	*Staphylococcus succinus 14BME20*	GCF_001902315.1
Dialister pneumosintes	GCF_001717505.1	*Staphylococcus vitulinus F1028*	GCF_000286335.1
Dialister succinatiphilus YIT 11850	GCF_000242435.1	*Staphylococcus warneri SG1*	GCF_000332735.1
Dielma fastidiosa	GCF_000313565.1	*Staphylococcus xylosus HKUOPL8*	GCF_000706685.1
Dietzia cinnamea	GCF_001643175.1	*Stenotrophomonas maltophilia K279a*	GCF_000072485.1
Dietzia maris	GCF_001630765.1	*Stenotrophomonas rhizophila QL-P4*	GCF_001704155.1
Dietzia natronolimnaea	GCF_002289575.1	*Streptococcus agalactiae 2603V/R*	GCF_000007265.1
Dorea formicigenerans ATCC 27755	GCF_000169235.1	*Streptococcus anginosus C238*	GCF_000463505.1
Dorea longicatena DSM 13814	GCF_000154065.1	*Streptococcus australis NCTC13166*	GCF_900476055.1
Dyadobacter beijingensis DSM 21582	GCF_000382205.1	*Streptococcus constellatus subsp. pharyngis C818*	GCF_000463445.1
Dyadobacter fermentans DSM 18053	GCF_000023125.1	*Streptococcus cristatus AS 1.3089*	GCF_000385925.1
Dysgonomonas gadei ATCC BAA-286	GCF_000213555.1	*Streptococcus dysgalactiae subsp. equisimilis AC-2713*	GCF_000317855.1
Dysgonomonas mossii DSM 22836	GCF_000213575.1	*Streptococcus equi subsp. Zooepidemicus H70*	GCF_000026605.1
Edwardsiella tarda FL6-60	GCF_000146305.1	*Streptococcus equinus AG46*	GCF_000964315.1
Eggerthella lenta DSM 2243	GCF_000024265.1	*Streptococcus gallolyticus subsp. gallolyticus DSM 16831*	GCF_002000985.1
Eggerthella sp. YY7918	GCF_000270285.1	*Streptococcus gordonii str. Challis substr. CH1*	GCF_000017005.1
Eggerthia catenaformis OT 569 = DSM 20559	GCF_000340375.1	*Streptococcus infantarius subsp. infantarius CJ18*	GCF_000246835.1
Empedobacter falsenii	GCF_003935845.1	*Streptococcus infantis ATCC 700779*	GCF_000187465.1
Enorma massiliensis phI	GCF_000311845.1	*Streptococcus intermedius B196*	GCF_000463355.1
Enterobacter asburiae	GCF_001521715.1	*Streptococcus lutetiensis 033*	GCF_000441535.1
Enterobacter cancerogenus	GCF_002850575.1	*Streptococcus mitis B6*	GCF_000027165.1
Enterobacter cloacae subsp. cloacae ATCC 13047	GCF_000025565.1	*Streptococcus mutans UA159*	GCF_000007465.2
Enterobacter hormaechei subsp. steigerwaltii	GCF_001729725.1	*Streptococcus oralis ATCC 35037*	GCF_900637025.1
Enterobacter ludwigii	GCF_001750725.1	*Streptococcus parasanguinis ATCC 15912*	GCF_000164675.2

Enterobacteriaceae bacterium w6	GCF_003336345.1	*Streptococcus parauberis KCTC 11537*	GCF_000213825.1
Enterococcus asini ATCC 700915	GCF_000407365.1	*Streptococcus pasteurianus ATCC 43144*	GCF_000270165.1
Enterococcus avium ATCC 14025	GCF_000407245.1	*Streptococcus peroris ATCC 700780*	GCF_000187585.1
Enterococcus caccae ATCC BAA-1240	GCF_000407145.1	*Streptococcus pneumoniae R6*	GCF_000007045.1
Enterococcus casseliflavus EC20	GCF_000157355.2	*Streptococcus pseudopneumoniae IS7493*	GCF_000221985.1
Enterococcus cecorum	GCF_001318405.1	*Streptococcus pyogenes M1 GAS*	GCF_000006785.2
Enterococcus dispar ATCC 51266	GCF_000406945.1	*Streptococcus salivarius NCTC 8618*	GCF_000785515.1
Enterococcus durans	GCF_001267865.1	*Streptococcus sanguinis SK36*	GCF_000014205.1
Enterococcus faecalis V583	GCF_000007785.1	*Streptococcus sp. oral taxon 064*	GCF_001683375.1
Enterococcus faecium DO	GCF_000174395.2	*Streptococcus thermophilus JIM 8232*	GCF_000253395.1
Enterococcus gallinarum FDAARGOS_163	GCF_001558875.2	*Streptococcus thoraltensis DSM 12221*	GCF_000380145.1
Enterococcus hirae ATCC 9790	GCF_000271405.2	*Streptococcus uberis 0140J*	GCF_000009545.1
Enterococcus phoeniculicola ATCC BAA-412	GCF_000407505.1	*Streptococcus vestibularis NCTC12167*	GCF_900636445.1
Enterococcus pseudoavium NBRC 100491	GCF_001544295.1	*Streptococcus viridans NCTC3166*	GCF_900636365.1
Enterococcus saccharolyticus subsp. saccharolyticus ATCC 43076	GCF_000407285.1	*Streptomyces misionensis DSM 40306*	GCF_900104815.1
Enterococcus sp. FDAARGOS_553	GCF_003812305.1	*Streptomyces thermovulgaris NRRL B-12375*	GCF_002155915.1
Erysipelatoclostridium ramosum DSM 1402	GCF_000154485.1	*Subdoligranulum sp. 4_3_54A2FAA*	GCF_000238635.1
Erysipelotrichaceae bacterium SG0102	GCF_003925875.1	*Subdoligranulum variabile DSM 15176*	GCF_000157955.1
Escherichia albertii NCTC 9362	GCF_003864095.1	*Succinatimonas hippei YIT 12066*	GCF_000188195.1
Escherichia coli O157:H7 str. Sakai	GCF_000008865.2	*Succinivibrio dextrinosolvens DSM 3072*	GCF_900167015.1
Escherichia fergusonii ECD227	GCF_000191665.1	*Sutterella parvirubra YIT 11816*	GCF_000250875.1
Escherichia sp. 1_1_43	GCF_000159895.2	*Sutterella wadsworthensis HGA0223*	GCF_000411515.1
Eubacterium barkeri VPI 5359	GCF_900107125.1	*Synergistes sp. 3_1_syn1*	GCF_000238615.1
Eubacterium brachy ATCC 33089	GCF_000488855.1	*Tannerella forsythia 92A2*	GCF_000238215.1
Eubacterium callanderi KIST612	GCF_000152245.2	*Tannerella sp. oral taxon HOT-286*	GCF_003033925.1
Eubacterium cellulosolvens 6	GCF_000183525.2	*Tatumella ptyseos NCTC11468*	GCF_900478715.1
Eubacterium eligens ATCC 27750	GCF_000146185.1	*Terrisporobacter glycolicus ATCC 14880 = DSM 1288*	GCF_000439105.1
Eubacterium hallii DSM 3353	GCF_000173975.1	*Tetragenococcus solitarius NBRC 100494*	GCF_001544195.1
Eubacterium limosum ATCC 8486	GCF_000807675.2	*Timonella senegalensis JC301*	GCF_000312125.1
Eubacterium ramulus ATCC 29099	GCF_000469345.1	*Tissierella praeacuta DSM 18095*	GCF_900128955.1
Eubacterium rectale ATCC 33656	GCF_000020605.1	*Trabulsiella guamensis ATCC 49490*	GCF_000734965.1
Eubacterium ruminantium ATCC 17233	GCF_900167085.1	*Treponema berlinense ATCC BAA-909*	GCF_900167025.1
Eubacterium saphenum ATCC 49989	GCF_000161975.1	*Tropheryma whipplei str. Twist*	GCF_000007485.1
Eubacterium siraeum DSM 15702	GCF_000382085.1	*Trueperella bernardiae LCDC 89-0504*	GCF_001469025.1
Eubacterium sp. 3_1_31	GCF_000242955.1	*Trueperella pyogenes TP6375*	GCF_000612055.1
Eubacterium sulci ATCC 35585	GCF_001189495.1	*Tumebacillus permanentifrigoris DSM 18773*	GCF_003148565.1
Eubacterium ventriosum ATCC 27560	GCF_000153885.1	*Turicibacter sanguinis 2789STDY5834851*	GCF_001406595.1
Eubacterium yurii subsp. margaretiae ATCC 43715	GCF_000146855.1	*Turicibacter sp. H121*	GCF_001543345.1
Exiguobacterium aurantiacum DSM 6208	GCF_000702585.1	*Tyzzerella nexilis DSM 1787*	GCF_000156035.2
Exiguobacterium sp. N4-1P	GCF_002214325.1	*Ureaplasma parvum serovar 3 str. ATCC 27815*	GCF_000019345.1
Faecalibacterium cf. prausnitzii KLE1255	GCF_000166035.1	*Ureaplasma urealyticum serovar 10 str. ATCC 33699*	GCF_000021265.1
Faecalibacterium prausnitzii A2-165	GCF_000162015.1	*Ureibacillus thermosphaericus str. Thermo-BF*	GCF_000284835.1
Faecalicoccus pleomorphus DSM 20574	GCF_000420345.1	*Varibaculum cambriense DSM 15806*	GCF_000420065.1
Faecalitalea cylindroides T2-87	GCF_000210615.1	*Veillonella atypica KON*	GCF_000318355.2
Fictibacillus arsenicus G25-54	GCF_001642935.1	*Veillonella dispar NCTC11831*	GCF_900637515.1
Filifactor alocis ATCC 35896	GCF_000163895.2	*Veillonella parvula DSM 2008*	GCF_000024945.1
Finegoldia magna ATCC 29328	GCF_000010185.1	*Veillonella ratti ATCC 17746*	GCF_003992115.1
Flavobacterium lindanitolerans DSM 21886	GCF_003663835.1	*Veillonella rogosae JCM 15642*	GCF_002959775.1
Flavobacterium oncorhynchi CCUG 59446	GCF_002217355.1	*Veillonella sp. AF13-2*	GCF_003603295.1
Flavobacterium sp. CJ74	GCF_003403075.1	*Vibrio cholerae O1 biovar El Tor str. N16961*	GCF_000006745.1
Flavonifractor plautii YL31	GCF_001688625.2	*Vibrio fluvialis ATCC 33809*	GCF_001558415.2
Fusicatenibacter saccharivorans 2789STDY5608849	GCF_001405555.1	*Vibrio furnissii NCTC 11218*	GCF_000184325.1
Fusobacterium gonidiaformans ATCC 25563	GCF_003019695.1	*Vibrio mimicus ATCC 33654*	GCF_001558475.2
Fusobacterium mortiferum ATCC 9817	GCF_000158195.2	*Vibrio parahaemolyticus RIMD 2210633*	GCF_000196095.1
Fusobacterium naviforme ATCC 25832	GCF_003014445.1	*Vibrio sp. HBUAS61001*	GCF_003716875.1
Fusobacterium necrogenes NCTC10723	GCF_900450765.1	*Victivallis vadensis DSM 14823*	GCF_003096415.1
Fusobacterium necrophorum subsp. funduliforme ATCC 51357	GCF_000262225.1	*Virgibacillus proomii V-P*	GCF_900162615.1
Fusobacterium nucleatum subsp. nucleatum ATCC 25586	GCF_000007325.1	*Weissella cibaria CH2*	GCF_001308145.2
Fusobacterium periodonticum 2_1_31	GCF_000158215.3	*Weissella confusa DSM 20196*	GCF_001436895.1

Fusobacterium russii ATCC 25533	GCF_000381725.1	*Weissella paramesenteroides ATCC 33313*	GCF_000160575.1
Fusobacterium sp. oral taxon 203 W7671	GCF_002243405.1	*Xenorhabdus nematophila AN6/1*	GCF_000953355.1
Fusobacterium ulcerans ATCC 49185	GCF_000158315.2	*Yersinia aleksiciae 159*	GCF_001047675.1
Fusobacterium varium ATCC 27725	GCF_003019655.1	*Yersinia bercovieri 127/84*	GCF_001131685.1
gamma proteobacterium HdN1	GCF_000198515.1	*Yersinia enterocolitica subsp. enterocolitica 8081*	GCF_000009345.1
Gardnerella vaginalis ATCC 14019	GCF_000159155.2	*Yersinia frederiksenii Y225*	GCF_000834215.1
Gemella haemolysans ATCC 10379	GCF_000173915.1	*Yersinia kristensenii ATCC BAA-2637*	GCF_003600645.1
Gemella morbillorum NCTC11323	GCF_900476045.1	*Yersinia pseudotuberculosis str. PA3606*	GCF_000834945.1
Gemella sanguinis ATCC 700632	GCF_000701685.1	*Yersinia rohdei YRA*	GCF_000834455.1
Gemmiger formicilis ATCC 27749	GCF_900167555.1	*Yokenella regensburgei ATCC 49455*	GCF_000735455.1

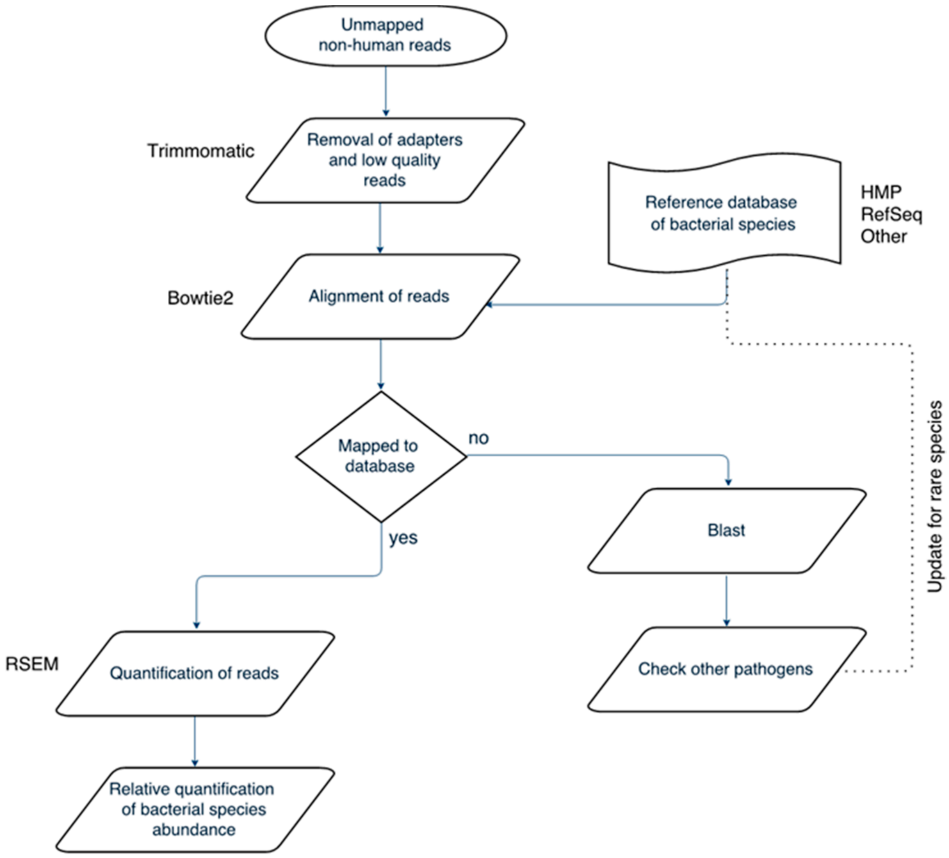

Supplementary Figure S1. Schematic representation of the QmihR pipeline [5].

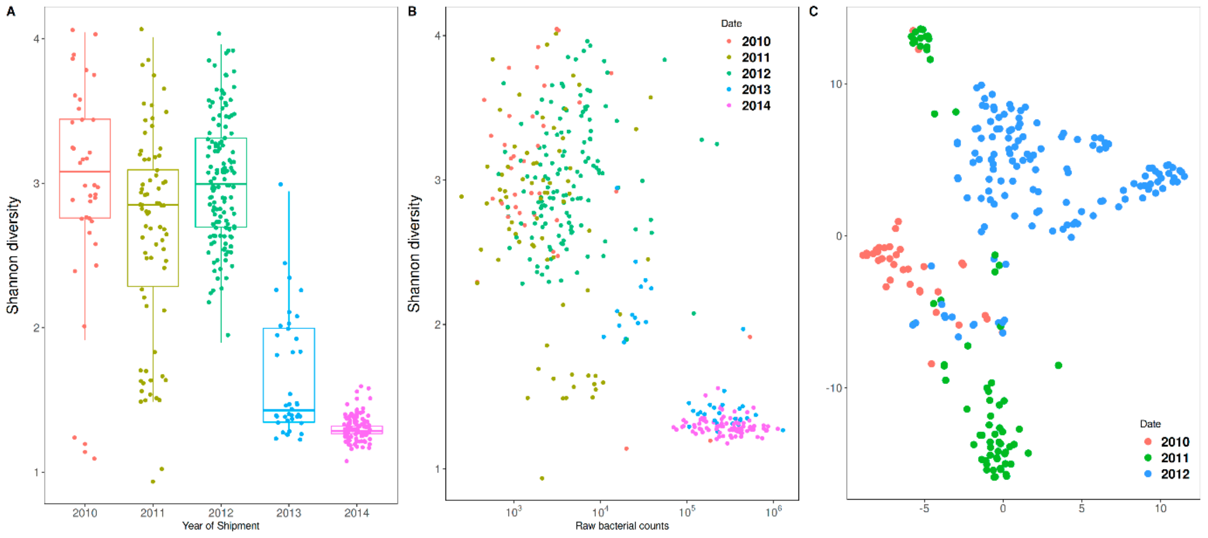

Supplementary Figure S2. Quality control assessment of the TCGA stomach adenocarcinoma samples based on the raw bacterial counts and alpha diversity (Shannon). (A) Boxplot distribution of the alpha diversity based on shipment year. (B) scatterplot of the distribution of raw reads according to the Shannon diversity index throughout the different shipment years. (C) T-distributed Stochastic Neighbour Embedding (t-SNE) plot based on the Weighted Unifrac distance between samples.

Supplementary Table S3. Percentage of abundance per sample of *Helicobacter* and of genera that reach a value of 90% (*Escherichia*, *Lactobacillus* and *Pseudomonas*), with information of country of origin and year of shipment.

Sample	Country	Date	*Escherichia*	*Helicobacter*	*Lactobacillus*	*Pseudomonas*
TCGA-CG-4301-01A	Germany	2010	25.34	0.00	0.00	19.83
TCGA-CG-4304-01A	Germany	2010	23.14	0.32	0.65	43.38
TCGA-CG-4305-01A	Germany	2010	31.16	0.00	0.12	9.88
TCGA-CG-4306-01A	Germany	2010	1.55	0.37	86.15	0.37
TCGA-CG-4436-01A	Germany	2010	9.39	0.03	1.25	2.28
TCGA-CG-4438-01A	Germany	2010	32.83	0.00	0.00	2.87
TCGA-CG-4440-01A	Germany	2010	26.78	0.00	0.08	32.44
TCGA-CG-4442-01A	Germany	2010	2.02	0.00	10.44	0.49
TCGA-CG-4443-01A	Germany	2010	33.39	0.00	3.15	4.37
TCGA-CG-4444-01A	Germany	2010	49.33	0.00	0.00	3.96
TCGA-CG-4460-01A	Germany	2010	15.61	0.00	0.18	5.32
TCGA-CG-4462-01A	Germany	2010	16.49	0.00	0.37	1.73
TCGA-CG-4465-01A	Germany	2010	11.52	0.00	0.05	3.92
TCGA-CG-4466-01A	Germany	2010	6.58	0.00	0.46	1.76
TCGA-CG-4469-01A	Germany	2010	16.19	0.00	0.34	15.52
TCGA-CG-4475-01A	Germany	2010	40.36	0.00	0.00	4.61
TCGA-CG-4476-01A	Germany	2010	36.24	0.00	0.10	4.80

TCGA-CG-4477-01A	Germany	2010	36.01	0.00	0.00	9.65
TCGA-BR-4187-01A	Russia	2010	1.68	3.25	22.33	7.18
TCGA-BR-4191-01A	Russia	2010	19.51	0.00	6.04	1.42
TCGA-BR-4201-01A	Russia	2010	20.26	0.00	0.22	3.10
TCGA-BR-4253-01A	Russia	2010	0.00	0.00	1.85	82.24
TCGA-BR-4256-01A	Russia	2010	34.63	0.00	1.23	16.93
TCGA-BR-4257-01A	Russia	2010	1.32	0.00	3.07	12.06
TCGA-BR-4267-01A	Russia	2010	0.05	0.00	0.05	0.50
TCGA-BR-4279-01A	Russia	2010	3.24	4.59	3.13	18.53
TCGA-BR-4280-01A	Russia	2010	19.31	0.85	0.35	2.56
TCGA-BR-4294-01A	Russia	2010	49.86	0.00	0.21	4.97
TCGA-BR-4357-01A	Russia	2010	56.73	1.06	0.00	5.28
TCGA-BR-4361-01A	Russia	2010	16.01	0.19	48.66	1.56
TCGA-BR-4363-01A	Russia	2010	0.00	0.00	0.07	97.51
TCGA-BR-4366-01A	Russia	2010	23.18	0.00	0.18	6.75
TCGA-BR-4367-01A	Russia	2010	16.53	0.27	0.23	3.13
TCGA-BR-4368-01A	Russia	2010	26.60	0.00	39.03	1.55
TCGA-BR-4369-01A	Russia	2010	25.20	0.05	0.38	8.28
TCGA-BR-4370-01A	Russia	2010	20.84	0.00	3.94	9.78
TCGA-BR-4371-01A	Russia	2010	13.81	0.03	0.86	14.29
TCGA-HF-7131-01A	Canada	2011	0.00	0.00	0.00	88.86
TCGA-HF-7132-01A	Canada	2011	0.00	0.00	0.20	92.82
TCGA-HF-7133-01A	Canada	2011	0.03	0.00	0.05	92.61
TCGA-HF-7134-01A	Canada	2011	0.05	0.19	0.05	81.13
TCGA-CG-4437-01A	Germany	2011	0.75	0.14	1.08	1.55
TCGA-CG-4441-01A	Germany	2011	0.27	0.00	2.86	2.59
TCGA-CG-5716-01A	Germany	2011	0.00	0.28	0.76	1.42
TCGA-CG-5717-01A	Germany	2011	0.16	0.00	0.54	6.70
TCGA-CG-5718-01A	Germany	2011	0.32	0.00	7.51	0.45
TCGA-CG-5719-01A	Germany	2011	2.17	0.12	0.82	5.89
TCGA-CG-5720-01A	Germany	2011	0.35	19.60	1.21	1.47
TCGA-CG-5721-01A	Germany	2011	1.68	0.00	14.40	1.56
TCGA-CG-5722-01A	Germany	2011	0.80	0.43	1.47	1.49
TCGA-CG-5723-01A	Germany	2011	0.03	0.06	0.25	0.15
TCGA-CG-5724-01A	Germany	2011	0.30	0.03	3.28	3.51
TCGA-CG-5725-01A	Germany	2011	0.93	0.12	1.86	2.21
TCGA-CG-5726-01A	Germany	2011	0.60	0.00	0.95	1.19
TCGA-CG-5732-01A	Germany	2011	0.08	0.00	0.16	43.30
TCGA-CG-5734-01A	Germany	2011	0.25	0.00	1.27	1.25
TCGA-D7-5577-01A	Poland	2011	0.32	0.00	0.83	5.47
TCGA-D7-5578-01A	Poland	2011	0.94	0.00	1.94	1.17
TCGA-D7-6519-01A	Poland	2011	0.00	0.00	0.00	1.41
TCGA-D7-6520-01A	Poland	2011	0.24	0.00	0.39	0.49
TCGA-D7-6521-01A	Poland	2011	0.63	0.50	1.64	1.39
TCGA-D7-6522-01A	Poland	2011	0.61	2.14	0.73	2.25
TCGA-D7-6524-01A	Poland	2011	0.00	0.00	8.46	1.30
TCGA-D7-6525-01A	Poland	2011	0.00	1.02	6.76	2.46
TCGA-D7-6526-01A	Poland	2011	0.57	0.00	66.48	0.98

TCGA-D7-6527-01A	Poland	2011	0.35	3.79	0.77	2.74
TCGA-D7-6528-01A	Poland	2011	0.72	0.00	3.61	1.59
TCGA-D7-6815-01A	Poland	2011	1.06	0.00	2.38	1.06
TCGA-D7-6818-01A	Poland	2011	0.21	0.00	5.91	0.28
TCGA-D7-6822-01A	Poland	2011	1.73	0.00	2.02	0.86
TCGA-B7-5818-01A	Russia	2011	0.02	0.21	6.77	0.07
TCGA-BR-6452-01A	Russia	2011	0.80	6.00	2.00	1.20
TCGA-BR-6453-01A	Russia	2011	0.89	0.74	0.98	1.42
TCGA-BR-6454-01A	Russia	2011	0.00	0.00	0.85	3.05
TCGA-BR-6455-01A	Russia	2011	0.00	0.12	0.33	7.66
TCGA-BR-6456-01A	Russia	2011	0.00	0.00	1.27	1.27
TCGA-BR-6563-01A	Russia	2011	0.00	0.23	0.05	89.61
TCGA-BR-6564-01A	Russia	2011	0.18	0.00	1.60	1.51
TCGA-BR-6565-01A	Russia	2011	0.00	0.00	0.24	0.72
TCGA-BR-6566-01A	Russia	2011	1.16	0.12	1.40	1.40
TCGA-BR-6801-01A	Russia	2011	0.21	0.02	27.06	0.24
TCGA-BR-6802-01A	Russia	2011	0.00	0.00	1.81	0.79
TCGA-BR-6803-01A	Russia	2011	0.62	64.67	0.00	0.97
TCGA-BR-7704-01A	Russia	2011	0.06	0.02	0.02	91.80
TCGA-BR-7707-01A	Russia	2011	0.08	0.00	1.13	89.03
TCGA-BR-7715-01A	Russia	2011	0.20	0.13	4.48	62.96
TCGA-BR-7716-01A	Russia	2011	0.10	0.18	0.04	77.14
TCGA-BR-7717-01A	Russia	2011	0.00	0.00	37.82	44.73
TCGA-BR-6457-01A	Ukraine	2011	0.00	0.00	2.42	1.16
TCGA-BR-6458-01A	Ukraine	2011	0.00	0.00	1.66	1.96
TCGA-BR-6705-01A	Ukraine	2011	0.34	0.00	0.23	1.02
TCGA-BR-6707-01A	Ukraine	2011	0.82	0.00	0.23	1.99
TCGA-BR-6709-01A	Ukraine	2011	0.00	0.35	1.05	5.07
TCGA-BR-6710-01A	Ukraine	2011	0.00	84.20	0.28	0.66
TCGA-BR-6852-01A	Ukraine	2011	0.00	0.00	0.52	0.34
TCGA-BR-7196-01A	Ukraine	2011	0.01	0.01	0.00	88.18
TCGA-BR-7723-01A	Ukraine	2011	0.10	0.00	0.10	88.97
TCGA-F1-6177-01A	United States	2011	0.01	0.00	3.73	0.09
TCGA-F1-6874-01A	United States	2011	0.29	0.10	9.47	0.39
TCGA-F1-6875-01A	United States	2011	0.17	0.00	0.04	88.40
TCGA-FP-7735-01A	United States	2011	0.05	0.00	0.07	90.71
TCGA-FP-7829-01A	United States	2011	0.29	0.00	0.04	84.61
TCGA-IN-7806-01A	United States	2011	0.08	0.00	0.02	91.70
TCGA-CD-5798-01A	Vietnam	2011	0.92	0.00	1.44	1.48
TCGA-CD-5799-01A	Vietnam	2011	0.75	0.00	1.67	0.75
TCGA-CD-5800-01A	Vietnam	2011	0.55	0.00	1.04	1.42
TCGA-CD-5801-01A	Vietnam	2011	0.00	0.00	1.80	1.67
TCGA-CD-5803-01A	Vietnam	2011	0.49	0.00	11.91	1.42
TCGA-CD-5804-01A	Vietnam	2011	0.03	0.00	0.01	88.47
TCGA-CD-5813-01A	Vietnam	2011	0.07	0.18	0.71	29.30
TCGA-D7-8570-01A	Poland	2012	0.00	0.00	0.00	35.94
TCGA-D7-8572-01A	Poland	2012	0.28	0.31	0.65	12.27
TCGA-D7-8573-01A	Poland	2012	0.56	2.92	0.31	29.56

TCGA-D7-8574-01A	Poland	2012	1.00	0.00	0.28	30.80
TCGA-D7-8575-01A	Poland	2012	3.54	0.00	0.87	23.96
TCGA-D7-8576-01A	Poland	2012	0.77	0.00	0.19	35.40
TCGA-D7-8578-01A	Poland	2012	3.28	0.03	12.00	26.36
TCGA-D7-8579-01A	Poland	2012	1.15	1.68	1.63	41.83
TCGA-D7-A4YU-01A	Poland	2012	0.65	0.00	0.39	10.59
TCGA-D7-A4YX-01A	Poland	2012	0.53	0.00	0.42	10.14
TCGA-D7-A4Z0-01A	Poland	2012	0.21	0.04	1.63	8.79
TCGA-BR-7851-01A	Russia	2012	0.03	0.02	11.52	5.85
TCGA-BR-7957-01A	Russia	2012	0.00	0.06	0.18	36.01
TCGA-BR-7958-01A	Russia	2012	1.07	0.00	0.23	27.81
TCGA-BR-7959-01A	Russia	2012	0.04	0.00	0.00	50.44
TCGA-BR-8058-01A	Russia	2012	1.63	0.00	0.19	33.27
TCGA-BR-8059-01A	Russia	2012	0.54	0.00	12.40	22.11
TCGA-BR-8060-01A	Russia	2012	0.02	0.00	0.42	1.94
TCGA-BR-8077-01A	Russia	2012	0.15	0.01	0.21	28.65
TCGA-BR-8080-01A	Russia	2012	1.20	0.02	0.29	24.90
TCGA-BR-8081-01A	Russia	2012	0.52	0.00	0.69	20.69
TCGA-BR-8284-01A	Russia	2012	0.00	0.00	0.14	36.71
TCGA-BR-8286-01A	Russia	2012	0.76	0.08	0.21	25.48
TCGA-BR-8289-01A	Russia	2012	0.08	0.00	0.06	15.92
TCGA-BR-8291-01A	Russia	2012	0.35	0.00	0.09	30.63
TCGA-BR-8295-01A	Russia	2012	0.15	0.00	0.15	32.90
TCGA-BR-8296-01A	Russia	2012	0.67	0.06	0.12	34.74
TCGA-BR-8297-01A	Russia	2012	0.00	0.07	0.14	24.15
TCGA-BR-8361-01A	Russia	2012	0.00	0.00	1.59	34.44
TCGA-BR-8364-01A	Russia	2012	0.00	0.38	0.00	24.74
TCGA-BR-8372-01A	Russia	2012	0.91	0.00	1.26	30.36
TCGA-BR-8484-01A	Russia	2012	0.00	0.00	0.34	31.54
TCGA-BR-8485-01A	Russia	2012	1.39	1.04	0.00	28.06
TCGA-BR-8486-01A	Russia	2012	0.06	3.98	0.67	10.04
TCGA-BR-8588-01A	Russia	2012	0.43	0.00	0.09	24.33
TCGA-BR-8589-01A	Russia	2012	0.11	0.00	0.91	25.27
TCGA-BR-8590-01A	Russia	2012	0.18	0.01	0.71	6.15
TCGA-BR-8591-01A	Russia	2012	1.72	0.00	60.26	9.55
TCGA-BR-8592-01A	Russia	2012	1.26	0.00	0.24	26.84
TCGA-BR-8678-01A	Russia	2012	1.36	0.05	0.50	15.33
TCGA-BR-8679-01A	Russia	2012	0.87	0.00	0.48	32.30
TCGA-BR-8687-01A	Russia	2012	0.47	0.47	0.24	28.35
TCGA-BR-A4CR-01A	Russia	2012	2.98	0.00	38.43	1.50
TCGA-BR-A4CS-01A	Russia	2012	0.58	0.02	0.35	9.14
TCGA-BR-A4PF-01A	Russia	2012	0.14	1.60	1.37	14.17
TCGA-HU-8238-01A	South Korea	2012	0.68	0.98	0.17	21.69
TCGA-HU-8244-01A	South Korea	2012	0.67	0.04	0.21	25.47
TCGA-HU-8602-01A	South Korea	2012	0.22	0.03	1.90	6.18
TCGA-HU-8604-01A	South Korea	2012	0.00	0.01	89.82	0.35
TCGA-HU-8608-01A	South Korea	2012	0.88	0.13	0.38	19.32
TCGA-HU-8610-01A	South Korea	2012	0.00	0.01	31.73	0.18

TCGA-HU-A4G2-01A	South Korea	2012	0.17	0.21	0.38	9.17
TCGA-HU-A4G3-01A	South Korea	2012	0.00	0.00	0.09	13.15
TCGA-HU-A4G8-01A	South Korea	2012	1.25	0.00	0.50	13.30
TCGA-HU-A4G9-01A	South Korea	2012	0.28	0.00	0.28	4.52
TCGA-HU-A4GC-01A	South Korea	2012	0.82	0.00	0.30	6.93
TCGA-HU-A4GF-01A	South Korea	2012	0.52	1.44	0.16	12.00
TCGA-HU-A4GH-01A	South Korea	2012	1.35	0.00	0.09	11.35
TCGA-HU-A4GJ-01A	South Korea	2012	0.34	6.11	0.20	8.88
TCGA-HU-A4GP-01A	South Korea	2012	0.35	0.00	0.76	7.47
TCGA-HU-A4GT-01A	South Korea	2012	0.71	0.00	2.00	7.48
TCGA-HU-A4GU-01A	South Korea	2012	0.18	0.00	19.99	2.54
TCGA-HU-A4GX-01A	South Korea	2012	0.60	0.00	0.75	10.93
TCGA-HU-A4GY-01A	South Korea	2012	1.35	0.03	0.21	16.57
TCGA-HU-A4H0-01A	South Korea	2012	1.14	0.00	0.08	8.17
TCGA-HU-A4H2-01A	South Korea	2012	0.23	0.20	60.80	4.06
TCGA-HU-A4H3-01A	South Korea	2012	0.02	0.15	38.21	0.20
TCGA-HU-A4H4-01A	South Korea	2012	1.78	0.07	0.37	14.17
TCGA-HU-A4H5-01A	South Korea	2012	0.51	0.11	0.81	8.77
TCGA-HU-A4H6-01A	South Korea	2012	0.35	0.12	50.55	4.36
TCGA-HU-A4H8-01A	South Korea	2012	1.23	0.06	0.12	17.46
TCGA-HU-A4HB-01A	South Korea	2012	1.13	0.27	7.35	17.80
TCGA-HU-A4HD-01A	South Korea	2012	0.32	5.49	0.29	7.35
TCGA-BR-7197-01A	Ukraine	2012	0.77	0.15	0.08	30.22
TCGA-BR-7722-01A	Ukraine	2012	0.00	0.00	0.27	32.73
TCGA-BR-7901-01A	Ukraine	2012	1.73	0.00	0.14	29.42
TCGA-BR-8365-01A	Ukraine	2012	0.00	0.00	0.16	34.00
TCGA-BR-8366-01A	Ukraine	2012	0.05	0.00	0.52	25.66
TCGA-BR-8367-01A	Ukraine	2012	0.16	0.00	0.49	27.95
TCGA-BR-8368-01A	Ukraine	2012	0.00	0.00	0.05	37.90
TCGA-BR-8369-01A	Ukraine	2012	1.23	0.29	0.51	40.20
TCGA-BR-8371-01A	Ukraine	2012	0.51	0.00	0.23	31.26
TCGA-BR-8373-01A	Ukraine	2012	3.74	0.90	0.00	34.53
TCGA-BR-8380-01A	Ukraine	2012	0.00	0.00	1.04	26.00
TCGA-BR-8381-01A	Ukraine	2012	0.79	0.04	0.25	21.30
TCGA-BR-8382-01A	Ukraine	2012	3.87	0.00	6.35	14.42
TCGA-BR-8384-01A	Ukraine	2012	0.43	0.11	0.05	14.42
TCGA-BR-8483-01A	Ukraine	2012	0.55	0.00	3.14	33.02
TCGA-BR-8487-01A	Ukraine	2012	1.08	0.00	1.97	20.83
TCGA-BR-8676-01A	Ukraine	2012	0.02	0.00	14.10	3.94
TCGA-BR-8677-01A	Ukraine	2012	0.05	0.00	0.61	16.36
TCGA-BR-8690-01A	Ukraine	2012	0.31	0.03	0.12	22.68
TCGA-BR-A44T-01A	Ukraine	2012	0.59	0.61	0.46	9.03
TCGA-BR-A4J6-01A	Ukraine	2012	0.24	0.00	0.13	6.61
TCGA-BR-A4J8-01A	Ukraine	2012	0.76	0.00	0.08	13.30
TCGA-BR-A4J9-01A	Ukraine	2012	0.61	0.00	0.27	9.49
TCGA-BR-A4QL-01A	Ukraine	2012	0.29	0.03	4.42	6.08
TCGA-EQ-8122-01A	United States	2012	1.14	0.03	0.13	38.66
TCGA-F1-A448-01A	United States	2012	2.08	0.00	1.53	14.41

TCGA-FP-7916-01A	United States	2012	0.62	0.00	0.22	30.29
TCGA-FP-7998-01A	United States	2012	0.90	0.00	0.00	38.72
TCGA-FP-8099-01A	United States	2012	0.97	0.00	0.04	32.04
TCGA-FP-8209-01A	United States	2012	0.42	0.00	0.15	33.22
TCGA-FP-8210-01A	United States	2012	0.86	0.00	0.23	34.07
TCGA-FP-8211-01A	United States	2012	0.00	0.00	0.13	25.21
TCGA-FP-8631-01A	United States	2012	1.12	0.00	0.00	27.56
TCGA-HJ-7597-01A	United States	2012	0.03	0.00	1.08	3.09
TCGA-IN-7808-01A	United States	2012	0.10	0.00	0.25	26.23
TCGA-IN-8462-01A	United States	2012	0.71	0.01	0.09	29.63
TCGA-IN-8663-01A	United States	2012	0.74	0.00	0.46	24.05
TCGA-IP-7968-01A	United States	2012	1.23	0.00	0.05	28.06
TCGA-BR-8680-01A	Vietnam	2012	0.68	0.00	0.19	51.06
TCGA-BR-8682-01A	Vietnam	2012	0.52	1.44	0.13	14.92
TCGA-BR-8683-01A	Vietnam	2012	0.05	0.00	13.31	6.77
TCGA-BR-8686-01A	Vietnam	2012	2.43	3.88	0.16	30.18
TCGA-BR-A4IV-01A	Vietnam	2012	0.51	0.02	0.37	9.99
TCGA-BR-A4J4-01A	Vietnam	2012	0.46	0.02	1.29	13.43
TCGA-BR-A4J5-01A	Vietnam	2012	0.09	0.01	0.18	5.25
TCGA-BR-A4J7-01A	Vietnam	2012	0.09	0.00	0.06	12.72
TCGA-CD-8524-01A	Vietnam	2012	0.28	0.00	1.36	33.13
TCGA-CD-8525-01A	Vietnam	2012	0.00	0.00	0.28	26.17
TCGA-CD-8526-01A	Vietnam	2012	0.33	0.00	0.37	28.56
TCGA-CD-8527-01A	Vietnam	2012	0.10	0.01	0.30	17.79
TCGA-CD-8528-01A	Vietnam	2012	0.97	0.06	0.19	40.91
TCGA-CD-8529-01A	Vietnam	2012	0.09	0.00	1.04	30.37
TCGA-CD-8530-01A	Vietnam	2012	0.08	0.00	0.10	32.51
TCGA-CD-8531-01A	Vietnam	2012	0.22	0.00	0.07	30.21
TCGA-CD-8532-01A	Vietnam	2012	0.30	0.00	0.09	11.78
TCGA-CD-8533-01A	Vietnam	2012	0.00	0.00	0.16	34.65
TCGA-CD-8534-01A	Vietnam	2012	0.35	0.00	0.26	38.55
TCGA-CD-8535-01A	Vietnam	2012	0.00	0.00	1.28	31.06
TCGA-CD-A486-01A	Vietnam	2012	0.00	0.19	1.78	11.96
TCGA-CD-A487-01A	Vietnam	2012	0.46	0.15	0.36	14.62
TCGA-CD-A489-01A	Vietnam	2012	0.64	0.00	0.42	11.46
TCGA-CD-A48C-01A	Vietnam	2012	0.33	0.00	11.63	12.82
TCGA-CD-A4MG-01A	Vietnam	2012	0.07	0.01	0.57	11.03
TCGA-CD-A4MH-01A	Vietnam	2012	0.80	0.05	0.10	11.39
TCGA-RD-A7BS-01A	Australia	2013	0.02	0.00	0.00	95.52
TCGA-RD-A7BT-01A	Australia	2013	0.02	0.00	0.00	96.67
TCGA-RD-A7BW-01A	Australia	2013	0.03	0.00	0.00	95.94
TCGA-RD-A7C1-01A	Australia	2013	0.02	0.00	0.00	97.07
TCGA-HF-A5NB-01A	Canada	2013	0.00	0.01	0.42	77.46
TCGA-KB-A6F7-01A	Canada	2013	0.00	0.00	0.00	96.67
TCGA-SW-A7EA-01A	Moldova	2013	0.04	0.00	0.00	95.85
TCGA-SW-A7EB-01A	Moldova	2013	0.00	0.00	0.00	94.84
TCGA-D7-A6EV-01A	Poland	2013	0.06	0.00	0.25	59.97
TCGA-D7-A6EX-01A	Poland	2013	0.02	0.00	0.03	79.56

TCGA-D7-A6EY-01A	Poland	2013	0.08	0.00	0.01	83.16
TCGA-D7-A6EZ-01A	Poland	2013	0.08	0.21	0.16	46.59
TCGA-D7-A6F0-01A	Poland	2013	0.27	0.00	0.01	83.57
TCGA-D7-A6F2-01A	Poland	2013	0.02	0.25	0.00	73.45
TCGA-D7-A747-01A	Poland	2013	0.00	0.00	0.00	96.03
TCGA-D7-A748-01A	Poland	2013	0.01	0.00	0.01	96.56
TCGA-D7-A74A-01A	Poland	2013	0.00	0.04	0.01	96.65
TCGA-B7-A5TI-01A	Russia	2013	0.04	0.02	0.20	73.94
TCGA-B7-A5TJ-01A	Russia	2013	0.19	0.00	0.07	79.61
TCGA-B7-A5TN-01A	Russia	2013	0.11	0.00	0.02	79.04
TCGA-F1-A72C-01A	United States	2013	0.01	0.00	0.00	97.73
TCGA-IN-A6RI-01A	United States	2013	0.01	0.00	0.00	97.19
TCGA-IN-A6RJ-01A	United States	2013	0.01	0.00	0.00	97.91
TCGA-IN-A6RL-01A	United States	2013	0.00	0.00	0.00	97.13
TCGA-IN-A6RN-01A	United States	2013	0.00	0.00	0.00	94.50
TCGA-IN-A6RO-01A	United States	2013	0.01	0.00	0.00	97.99
TCGA-IN-A6RR-01A	United States	2013	0.01	0.00	0.47	95.64
TCGA-IN-A6RS-01A	United States	2013	0.02	0.00	0.00	82.46
TCGA-IN-A7NR-01A	United States	2013	0.01	0.00	0.00	95.75
TCGA-IN-A7NT-01A	United States	2013	0.00	0.00	0.00	97.32
TCGA-IN-A7NU-01A	United States	2013	0.00	0.00	0.03	95.35
TCGA-MX-A5UG-01A	United States	2013	0.07	0.03	0.02	78.98
TCGA-MX-A5UJ-01A	United States	2013	0.12	0.00	0.00	79.10
TCGA-MX-A663-01A	United States	2013	0.01	0.00	0.04	81.42
TCGA-MX-A666-01A	United States	2013	0.13	0.00	0.02	83.79
TCGA-R5-A7O7-01A	United States	2013	0.00	0.00	0.11	95.11
TCGA-R5-A7ZE-01B	United States	2013	0.03	0.00	0.01	93.52
TCGA-R5-A7ZF-01A	United States	2013	0.01	0.00	0.09	96.53
TCGA-R5-A7ZI-01A	United States	2013	0.01	0.00	0.03	97.08
TCGA-R5-A7ZR-01A	United States	2013	0.02	0.00	0.00	94.70
TCGA-RD-A8MV-01A	Australia	2014	0.01	0.00	0.01	97.68
TCGA-RD-A8MW-01A	Australia	2014	0.01	0.00	0.00	97.99
TCGA-RD-A8N0-01A	Australia	2014	0.00	0.04	0.00	97.58
TCGA-RD-A8N1-01A	Australia	2014	0.02	0.00	0.02	98.02
TCGA-RD-A8N2-01A	Australia	2014	0.01	0.00	0.00	96.34
TCGA-RD-A8N4-01A	Australia	2014	0.01	0.00	0.00	97.19
TCGA-RD-A8N5-01A	Australia	2014	0.04	0.02	0.01	95.88
TCGA-RD-A8N6-01A	Australia	2014	0.03	0.00	0.00	97.55
TCGA-RD-A8N9-01A	Australia	2014	0.00	0.06	0.00	98.24
TCGA-RD-A8NB-01A	Australia	2014	0.01	0.00	0.00	97.98
TCGA-VQ-A8DT-01A	Brazil	2014	0.00	0.04	0.00	97.47
TCGA-VQ-A8DU-01A	Brazil	2014	0.01	0.00	0.00	97.17
TCGA-VQ-A8DV-01A	Brazil	2014	0.00	0.00	0.00	97.97
TCGA-VQ-A8DZ-01A	Brazil	2014	0.00	0.00	0.00	97.52
TCGA-VQ-A8E0-01A	Brazil	2014	0.01	0.00	0.07	98.04
TCGA-VQ-A8E2-01A	Brazil	2014	0.00	0.00	0.00	98.47
TCGA-VQ-A8E3-01A	Brazil	2014	0.00	0.00	0.00	97.74
TCGA-VQ-A8E7-01B	Brazil	2014	0.00	0.00	0.03	96.94

TCGA-VQ-A8P2-01A	Brazil	2014	0.04	0.00	0.01	96.49
TCGA-VQ-A8P3-01A	Brazil	2014	0.00	0.00	0.00	97.49
TCGA-VQ-A8P5-01A	Brazil	2014	0.00	0.00	0.00	95.21
TCGA-VQ-A8P8-01A	Brazil	2014	0.00	0.01	0.00	98.41
TCGA-VQ-A8PB-01A	Brazil	2014	0.07	0.00	0.24	97.72
TCGA-VQ-A8PC-01A	Brazil	2014	0.01	0.00	0.01	97.33
TCGA-VQ-A8PD-01A	Brazil	2014	0.00	0.00	0.12	98.46
TCGA-VQ-A8PE-01A	Brazil	2014	0.01	0.01	0.00	98.07
TCGA-VQ-A8PF-01A	Brazil	2014	0.00	0.00	0.01	97.96
TCGA-VQ-A8PH-01A	Brazil	2014	0.00	0.00	0.24	97.74
TCGA-VQ-A8PJ-01A	Brazil	2014	0.01	0.00	0.00	97.60
TCGA-VQ-A8PK-01A	Brazil	2014	0.00	0.00	0.01	96.55
TCGA-VQ-A8PM-01A	Brazil	2014	0.00	0.00	0.00	97.92
TCGA-VQ-A8PO-01A	Brazil	2014	0.02	0.00	0.02	96.89
TCGA-VQ-A8PP-01A	Brazil	2014	0.03	0.00	0.01	98.21
TCGA-VQ-A8PQ-01A	Brazil	2014	0.00	0.00	0.00	97.73
TCGA-VQ-A8PU-01A	Brazil	2014	0.01	0.00	0.00	97.14
TCGA-VQ-A8PX-01A	Brazil	2014	0.00	0.00	0.00	97.20
TCGA-VQ-A91A-01A	Brazil	2014	0.00	0.00	0.00	98.56
TCGA-VQ-A91D-01A	Brazil	2014	0.00	0.00	0.16	92.58
TCGA-VQ-A91E-01A	Brazil	2014	0.01	0.00	0.36	97.96
TCGA-VQ-A91K-01A	Brazil	2014	0.00	0.00	0.01	97.36
TCGA-VQ-A91N-01A	Brazil	2014	0.00	0.00	0.00	98.17
TCGA-VQ-A91Q-01A	Brazil	2014	0.00	0.00	0.00	97.91
TCGA-VQ-A91S-01A	Brazil	2014	0.00	0.00	0.00	98.12
TCGA-VQ-A91U-01A	Brazil	2014	0.00	0.39	0.03	97.69
TCGA-VQ-A91V-01A	Brazil	2014	0.00	0.00	0.00	98.40
TCGA-VQ-A91X-01A	Brazil	2014	0.00	0.01	0.00	98.56
TCGA-VQ-A91Y-01A	Brazil	2014	0.01	0.00	0.02	95.74
TCGA-VQ-A91Z-01A	Brazil	2014	0.00	0.00	0.00	97.18
TCGA-VQ-A922-01A	Brazil	2014	0.00	0.00	0.02	97.68
TCGA-VQ-A923-01A	Brazil	2014	0.00	0.02	0.01	97.29
TCGA-VQ-A924-01A	Brazil	2014	0.00	0.00	0.00	97.64
TCGA-VQ-A925-01A	Brazil	2014	0.00	0.00	0.01	98.09
TCGA-VQ-A927-01A	Brazil	2014	0.00	0.00	0.02	98.13
TCGA-VQ-A928-01A	Brazil	2014	0.01	0.00	0.11	97.67
TCGA-VQ-A92D-01A	Brazil	2014	0.02	0.00	0.00	97.40
TCGA-VQ-A94O-01A	Brazil	2014	0.01	0.00	0.00	97.85
TCGA-VQ-A94P-01A	Brazil	2014	0.00	0.00	0.34	97.57
TCGA-VQ-A94R-01A	Brazil	2014	0.00	0.00	0.32	96.56
TCGA-VQ-A94T-01A	Brazil	2014	0.01	0.04	0.00	98.11
TCGA-VQ-A94U-01A	Brazil	2014	0.00	0.00	0.00	98.36
TCGA-VQ-AA64-01A	Brazil	2014	0.01	0.00	0.00	97.67
TCGA-VQ-AA68-01A	Brazil	2014	0.02	0.00	0.00	97.92
TCGA-VQ-AA69-01A	Brazil	2014	0.00	0.00	0.00	93.26
TCGA-VQ-AA6A-01A	Brazil	2014	0.00	0.02	0.00	97.24
TCGA-VQ-AA6D-01A	Brazil	2014	0.00	0.00	0.00	96.80
TCGA-VQ-AA6F-01A	Brazil	2014	0.00	0.00	0.01	97.14

TCGA-VQ-AA6G-01A	Brazil	2014	0.00	0.00	0.00	97.79
TCGA-VQ-AA6J-01A	Brazil	2014	0.00	0.04	0.00	98.48
TCGA-VQ-AA6K-01A	Brazil	2014	0.00	0.00	0.00	97.60
TCGA-KB-A93G-01A	Canada	2014	0.00	0.00	0.00	97.64
TCGA-KB-A93H-01A	Canada	2014	0.00	0.00	0.01	96.91
TCGA-KB-A93J-01A	Canada	2014	0.01	0.00	0.03	97.32
TCGA-B7-A5TK-01A	Russia	2014	0.00	0.00	0.01	96.44
TCGA-HU-8249-01A	South Korea	2014	0.00	0.00	0.00	98.00
TCGA-HU-A4GD-01A	South Korea	2014	0.01	0.00	0.00	98.62
TCGA-HU-A4GQ-01A	South Korea	2014	0.00	0.00	0.03	96.92
TCGA-BR-A44U-01A	Ukraine	2014	0.01	0.00	0.00	97.51
TCGA-ZQ-A9CR-01A	United Kingdom	2014	0.00	0.00	0.00	97.58
TCGA-3M-AB46-01A	United States	2014	0.01	0.00	0.11	98.09
TCGA-3M-AB47-01A	United States	2014	0.00	0.00	0.00	98.67
TCGA-FP-A4BF-01A	United States	2014	0.00	0.00	0.00	97.63
TCGA-FP-A8CX-01A	United States	2014	0.01	0.00	0.21	95.08
TCGA-FP-A9TM-01A	United States	2014	0.01	0.00	0.00	98.22
TCGA-IN-AB1V-01A	United States	2014	0.01	0.00	0.00	97.20
TCGA-IN-AB1X-01A	United States	2014	0.01	0.00	0.00	97.08
TCGA-R5-A805-01A	United States	2014	0.01	0.00	0.00	95.24
TCGA-ZA-A8F6-01A	United States	2014	0.00	0.00	0.00	97.95
TCGA-CD-A48A-01A	Vietnam	2014	0.00	0.00	0.00	97.85
GTEX-O5YW-1526-SM-3MJGL	United States	2011	1.99	0.00	2.52	14.79
GTEX-OIZH-1526-SM-3NB1J	United States	2011	2.14	0.00	0.00	22.39
GTEX-OXRK-1626-SM-3NB17	United States	2011	3.33	0.00	0.00	13.58
GTEX-P4PP-1526-SM-3P61M	United States	2011	0.43	0.02	0.33	10.35
GTEX-P4PQ-1526-SM-3NMCK	United States	2011	14.08	0.00	2.68	12.82
GTEX-P4QT-1526-SM-3NMCT	United States	2011	14.98	0.00	0.80	31.19
GTEX-P78B-1826-SM-3P5YX	United States	2011	1.42	0.00	0.14	10.91
GTEX-PLZ6-0826-SM-3P61K	United States	2011	2.08	0.06	1.83	24.28
GTEX-PW2O-1226-SM-48TCH	United States	2011	6.63	0.00	3.54	25.39
GTEX-PWCY-0926-SM-48TD7	United States	2011	1.20	0.00	1.64	30.12
GTEX-PWN1-1526-SM-48TDA	United States	2011	1.91	0.00	2.11	29.05
GTEX-PWOO-1226-SM-48TCO	United States	2011	2.26	0.45	1.76	22.32
GTEX-PX3G-1526-SM-48U11	United States	2011	3.14	0.00	0.16	32.59
GTEX-Q2AI-0826-SM-48TZO	United States	2011	0.43	0.01	0.46	15.83
GTEX-Q734-1026-SM-48U16	United States	2011	0.76	0.00	0.04	18.94
GTEX-QCQG-0526-SM-48U2A	United States	2011	1.60	0.05	1.46	21.03
GTEX-QDVJ-1426-SM-48U1Y	United States	2011	2.40	0.00	1.39	45.66
GTEX-QDVN-1226-SM-48TZ5	United States	2011	2.11	0.00	1.62	20.79
GTEX-QMRM-1126-SM-447BN	United States	2011	0.50	0.02	0.13	5.16
GTEX-QV31-0626-SM-447C5	United States	2011	0.85	0.00	0.20	29.23
GTEX-QV44-1226-SM-4R1KE	United States	2011	35.91	0.00	5.73	5.61
GTEX-QXCU-1926-SM-48FE4	United States	2011	0.45	0.00	20.59	7.51
GTEX-R53T-1226-SM-48FCT	United States	2012	0.75	0.00	22.95	6.09
GTEX-R55C-1026-SM-48FCM	United States	2012	0.07	0.00	15.91	4.04
GTEX-R55G-1126-SM-48FDG	United States	2012	0.28	0.00	17.01	5.83

GTEX-RM2N-0826-SM-48FD3	United States	2012	0.27	0.00	20.75	7.05
GTEX-RTLS-2626-SM-46MUJ	United States	2012	1.71	0.00	0.15	21.91
GTEX-RWS6-0926-SM-47JXE	United States	2012	0.45	0.00	51.53	5.37
GTEX-S3XE-1026-SM-4AD4O	United States	2012	0.71	0.12	16.44	9.41
GTEX-S4P3-0726-SM-4AD57	United States	2012	0.56	0.01	4.56	2.65
GTEX-S4Q7-0726-SM-4AD5F	United States	2012	0.37	0.01	25.42	8.24
GTEX-S4UY-1626-SM-4AD55	United States	2012	0.24	0.11	13.8	10.26
GTEX-S4Z8-1226-SM-4AD6W	United States	2012	0.40	0.00	20.38	8.17
GTEX-S7SF-0626-SM-4AD4V	United States	2012	0.18	0.03	8.53	9.82
GTEX-S95S-0826-SM-4B64N	United States	2012	0.22	0.05	3.97	14.03
GTEX-SIU7-1426-SM-4BRWT	United States	2012	0.61	0.00	5.47	7.52
GTEX-SNMC-0626-SM-4DM6H	United States	2012	1.87	0.00	8.34	9.78
GTEX-SUCS-0926-SM-4DM4Z	United States	2012	0.16	0.01	5.89	8.79
GTEX-T5JC-1926-SM-4DM6Q	United States	2012	0.57	0.00	7.64	9.51
GTEX-T5JW-0926-SM-4DM5K	United States	2012	0.52	0.00	10.88	12.69
GTEX-T6MO-0726-SM-4DM58	United States	2012	0.63	0.00	7.08	7.68
GTEX-T8EM-1226-SM-4DM5J	United States	2012	1.18	0.00	8.54	10.38
GTEX-TKQ2-0926-SM-4DXU5	United States	2012	1.11	0.00	4.70	17.44
GTEX-TMMY-1626-SM-4DXTY	United States	2012	1.53	0.00	6.98	14.96
GTEX-U3ZN-2026-SM-4DXUC	United States	2012	0.74	0.00	2.71	12.35
GTEX-U4B1-1026-SM-4DXT1	United States	2012	0.31	0.01	1.75	2.85
GTEX-U8T8-1226-SM-4E3IH	United States	2012	3.64	0.00	1.25	2.10
GTEX-UPIC-1826-SM-4IHKC	United States	2012	25.64	0.00	0.98	9.16
GTEX-UPK5-2126-SM-4JBJK	United States	2012	26.96	0.00	1.98	9.67
GTEX-V1D1-1726-SM-4JBHB	United States	2012	30.15	0.00	0.81	12.16
GTEX-V955-1326-SM-4JBHR	United States	2012	27.25	0.00	0.87	8.86
GTEX-W5WG-1726-SM-4LMI5	United States	2012	23.26	0.00	0.53	4.90
GTEX-WEY5-1226-SM-4LMIQ	United States	2012	17.26	0.03	0.14	4.38
GTEX-WFG7-1326-SM-4LMK1	United States	2012	14.20	0.00	0.08	3.89
GTEX-WFG8-1326-SM-4LVN3	United States	2012	38.90	0.00	0.08	9.98
GTEX-WFJO-1126-SM-4LVLZ	United States	2012	27.59	0.06	0.67	6.78
GTEX-WFON-1126-SM-4LVMA	United States	2012	6.34	0.01	0.45	2.79
GTEX-WH7G-1526-SM-4LVMX	United States	2012	26.80	0.00	0.74	7.20
GTEX-WHPG-0426-SM-4M1XW	United States	2012	26.88	0.22	1.08	6.51
GTEX-WHSB-1226-SM-4M1XR	United States	2012	26.93	0.06	1.60	6.24
GTEX-WHWD-1426-SM-4OORU	United States	2012	27.99	0.00	1.07	7.64
GTEX-WRHK-0626-SM-4MVOE	United States	2012	24.05	0.00	0.19	4.94
GTEX-WY7C-1726-SM-4ONCC	United States	2012	44.27	0.00	0.19	9.49
GTEX-WYJK-2526-SM-4ONDF	United States	2012	45.66	0.00	0.04	8.75
GTEX-WYVS-1326-SM-4ONCQ	United States	2012	46.70	0.00	0.04	9.40
GTEX-WZTO-2126-SM-4PQYW	United States	2012	16.81	0.00	5.26	3.97
GTEX-X15G-1126-SM-4PQZG	United States	2012	26.87	0.00	5.64	5.14
GTEX-XAJ8-0326-SM-47JYI	United States	2012	0.45	0.00	46.82	5.17
GTEX-XBED-1226-SM-4AT5V	United States	2012	0.46	0.00	11.28	7.46
GTEX-XBEW-1826-SM-4RTWX	United States	2012	16.79	0.03	2.39	4.06
GTEX-XPVG-1526-SM-4B66C	United States	2012	0.10	0.02	2.05	10.05

GTEX-XQ8I-2026-SM-4BOOL	United States	2012	0.77	0.00	3.29	8.36
GTEX-XUJ4-1226-SM-4BOPD	United States	2012	0.60	0.81	4.79	10.47
GTEX-XUZC-0726-SM-4BOPH	United States	2012	0.07	0.01	13.49	2.27
GTEX-XV7Q-1326-SM-4BRWM	United States	2012	1.95	0.00	8.38	7.84
GTEX-XXEK-0826-SM-4BRWG	United States	2012	0.39	0.01	7.10	4.71
GTEX-111CU-0926-SM-5EGIK	United States	2013	4.52	0.00	0.25	11.06
GTEX-111YS-1126-SM-5GZYQ	United States	2013	1.26	0.00	0.46	7.69
GTEX-1122O-1926-SM-5EGIQ	United States	2013	1.20	0.00	0.06	18.69
GTEX-117YW-2226-SM-5N9DB	United States	2013	1.00	0.00	1.25	5.99
GTEX-11DXX-1326-SM-5GIDZ	United States	2013	1.97	4.82	0.11	12.50
GTEX-11EM3-1026-SM-5A5KL	United States	2013	3.00	0.00	1.67	10.00
GTEX-11EQ9-1226-SM-5987E	United States	2013	17.17	0.00	0.20	8.82
GTEX-11GSP-2126-SM-5HL5E	United States	2013	2.27	0.00	2.51	13.96
GTEX-11I78-1726-SM-5A5M3	United States	2013	2.22	0.00	3.39	9.66
GTEX-11NSD-1426-SM-5HL67	United States	2013	0.00	4.88	6.26	2.39
GTEX-11NUK-2426-SM-5BC4U	United States	2013	10.63	0.00	1.02	6.19
GTEX-11TT1-0726-SM-5GU5A	United States	2013	0.03	0.05	9.03	5.26
GTEX-11VI4-0326-SM-5EQ6L	United States	2013	0.62	0.00	0.35	15.85
GTEX-11WQK-2626-SM-5EQ4K	United States	2013	6.06	0.00	1.18	12.96
GTEX-1211K-1426-SM-5FQTF	United States	2013	1.34	0.00	0.33	7.31
GTEX-12696-1726-SM-5EQLH	United States	2013	0.37	0.12	0.33	6.66
GTEX-12BJ1-1726-SM-5HL9B	United States	2013	1.27	0.00	0.84	8.28
GTEX-12C56-0526-SM-5FQST	United States	2013	0.34	0.10	0.34	4.85
GTEX-12WS9-1426-SM-5FQT3	United States	2013	3.65	0.00	0.24	4.81
GTEX-12WSG-2026-SM-5FQUU	United States	2013	0.87	0.14	0.09	9.04
GTEX-12WSJ-0926-SM-5P9JD	United States	2013	0.36	0.00	0.57	4.91
GTEX-12WSK-1426-SM-5CVNN	United States	2013	3.89	0.00	1.37	10.98
GTEX-13111-1226-SM-5GCNC	United States	2013	1.81	0.00	2.09	13.25
GTEX-131XG-1226-SM-5EGH9	United States	2013	0.00	0.00	0.39	16.80
GTEX-132AR-2426-SM-5IFFD	United States	2013	1.72	0.00	0.94	10.47
GTEX-1339X-1426-SM-5K7YO	United States	2013	0.44	0.05	3.93	2.57
GTEX-133LE-1326-SM-5IFGO	United States	2013	1.52	0.00	0.51	18.73
GTEX-1399R-1126-SM-5IFIO	United States	2013	0.27	0.00	19.48	11.88
GTEX-139D8-2326-SM-5IFGE	United States	2013	1.69	0.02	0.37	8.02
GTEX-139YR-1926-SM-5LZXM	United States	2013	2.99	0.06	10.19	8.44
GTEX-13CF3-1926-SM-5K7WF	United States	2013	0.15	0.04	3.09	3.01
GTEX-13D11-2126-SM-5IFH2	United States	2013	0.54	0.00	0.31	3.99
GTEX-13FH7-1026-SM-5IJGF	United States	2013	0.25	0.00	1.41	3.03
GTEX-13FTW-1726-SM-5KM2B	United States	2013	0.34	0.00	0.67	4.32
GTEX-13FTX-0726-SM-5N9BI	United States	2013	0.62	0.00	0.00	7.37
GTEX-13N1W-2026-SM-5K7YU	United States	2013	0.28	0.11	2.79	4.29
GTEX-13O21-0926-SM-5IFGT	United States	2013	3.09	0.00	1.36	13.47
GTEX-13O3Q-2126-SM-5KM4C	United States	2013	1.71	0.04	1.67	9.77
GTEX-13OW6-2526-SM-5IJEC	United States	2013	1.01	0.00	0.19	13.67
GTEX-13PVQ-2126-SM-5L3FW	United States	2013	3.36	1.53	1.53	22.63
GTEX-13QBU-1126-SM-5LU44	United States	2013	0.32	0.00	1.68	17.92

GTEX-13U4I-2526-SM-5SI8Z	United States	2013	0.00	0.00	0.41	15.20
GTEX-144GM-1726-SM-5O9AS	United States	2013	0.12	0.01	0.15	4.74
GTEX-144GN-1226-SM-5O991	United States	2013	0.26	0.00	0.34	41.93
GTEX-145LT-1426-SM-5O9B3	United States	2013	1.6	0.00	0.40	6.82
GTEX-146FH-2126-SM-5SI9U	United States	2013	2.51	0.00	1.01	18.09
GTEX-146FR-2026-SM-5NQAI	United States	2013	0.07	0.00	2.00	13.99
GTEX-Y3I4-1626-SM-4TT7W	United States	2013	30.24	0.00	1.41	9.25
GTEX-Y5V6-1326-SM-4VDTF	United States	2013	29.96	0.00	0.27	6.37
GTEX-Y8E4-1526-SM-4WWDI	United States	2013	32.66	0.00	0.35	8.28
GTEX-Y8LW-0826-SM-4WWDO	United States	2013	39.93	0.00	0.30	9.40
GTEX-Y9LG-1026-SM-5IFJN	United States	2013	0.28	0.00	12.54	3.10
GTEX-YEC3-1426-SM-5PNXW	United States	2013	0.00	0.00	2.32	3.26
GTEX-YEC4-1426-SM-5IFHS	United States	2013	0.25	0.00	32.31	4.62
GTEX-YJ8A-1526-SM-5P9FT	United States	2013	1.09	0.00	1.00	15.40
GTEX-ZA64-0826-SM-5HL9U	United States	2013	1.11	0.00	1.82	7.66
GTEX-ZAB4-1526-SM-5CVN7	United States	2013	4.59	0.00	0.53	18.55
GTEX-ZDYS-1926-SM-5HL59	United States	2013	1.09	0.00	1.63	17.98
GTEX-ZE7O-3026-SM-51MS4	United States	2013	5.84	0.00	11.72	1.87
GTEX-ZEX8-1626-SM-4WKG7	United States	2013	38.71	0.00	0.16	9.35
GTEX-ZF29-1526-SM-4WKF7	United States	2013	22.01	0.00	0.15	7.37
GTEX-ZF2S-1426-SM-57WET	United States	2013	26.57	0.00	6.34	7.35
GTEX-ZLV1-0826-SM-4WWEQ	United States	2013	27.25	0.04	1.16	8.10
GTEX-ZP4G-1226-SM-4WWCJ	United States	2013	32.02	0.00	0.44	7.72
GTEX-ZPIC-2026-SM-57WG3	United States	2013	32.86	0.00	0.79	8.28
GTEX-ZT9W-1526-SM-4YCDE	United States	2013	30.14	0.10	1.03	8.19
GTEX-ZV6S-0926-SM-57WGB	United States	2013	11.29	0.00	0.64	4.07
GTEX-ZVT2-1626-SM-51MRC	United States	2013	40.52	0.00	0.22	9.23
GTEX-ZVT3-2126-SM-59HL2	United States	2013	19.02	0.00	3.84	15.75
GTEX-ZVZP-1726-SM-5GZWY	United States	2013	0.01	0.00	0.07	75.41
GTEX-ZXES-1426-SM-5NQ8S	United States	2013	0.93	0.00	1.04	9.11
GTEX-ZYFG-1326-SM-5GICJ	United States	2013	5.14	0.00	0.00	24.90
GTEX-ZYVF-2726-SM-5GID4	United States	2013	1.23	0.00	0.24	17.02
GTEX-ZYY3-1726-SM-5EGH3	United States	2013	1.51	0.00	0.39	5.20
GTEX-ZZ64-0426-SM-5E43F	United States	2013	1.37	0.00	0.29	10.45
GTEX-ZZPU-1426-SM-5GZZ6	United States	2013	0.00	0.00	0.61	12.84
GTEX-11EI6-2426-SM-5PNVS	United States	2014	0.32	0.00	2.90	3.75
GTEX-11P82-0726-SM-5PNYL	United States	2014	0.05	0.00	0.50	2.57
GTEX-1399S-1626-SM-5P9GI	United States	2014	1.70	0.00	0.01	9.48
GTEX-1399U-1626-SM-5P9J3	United States	2014	0.03	0.00	0.35	78.50
GTEX-13QJ3-2726-SM-5SI6L	United States	2014	0.65	0.01	0.80	7.43
GTEX-13RTK-0226-SM-5RQHR	United States	2014	5.62	0.00	1.18	30.18
GTEX-13S7M-1826-SM-5RQK6	United States	2014	0.92	0.00	1.37	16.59
GTEX-13X6H-1626-SM-5Q5CT	United States	2014	1.13	0.00	0.97	8.95
GTEX-145MO-2226-SM-5Q5BN	United States	2014	0.32	0.00	0.28	2.40
GTEX-14BMU-1126-SM-5RQJ8	United States	2014	2.66	0.00	0.35	17.91
GTEX-14BMV-2226-SM-5RQHX	United States	2014	0.16	0.02	0.29	14.71

GTEX-Y5LM-1326-SM-5RQIS	United States	2014	1.11	0.00	3.33	21.67

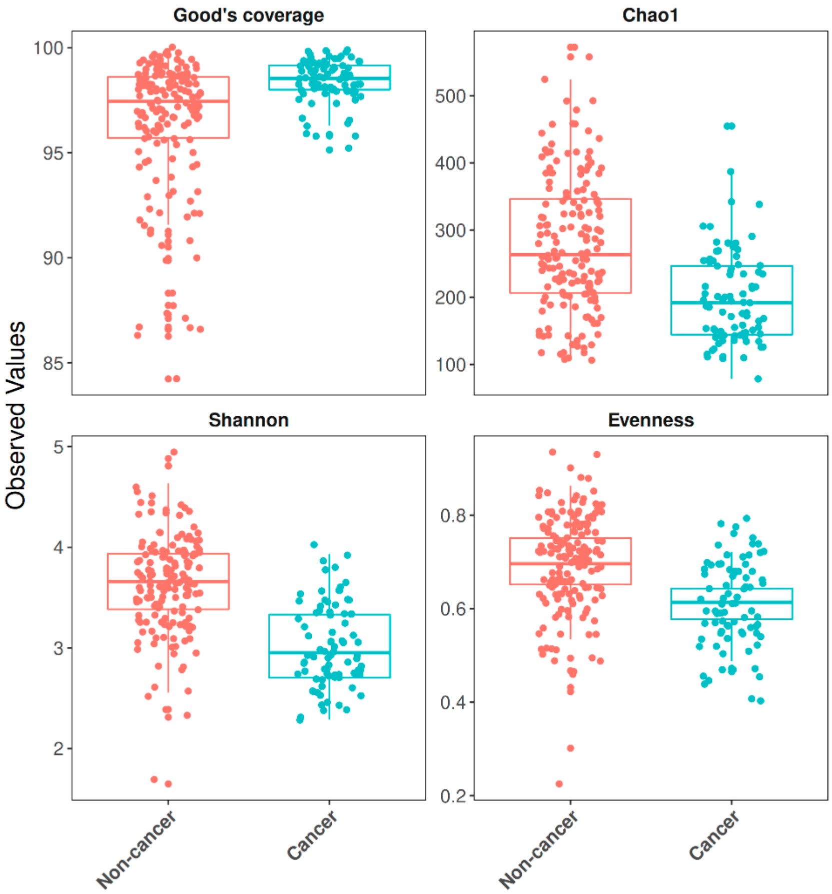

Supplementary Figure S3. Genus richness in the inferred microbiome patterns between the GTEx non-cancer samples and the TCGA cancer samples from the sample ancestral background (European and United states). A- Good's coverage, Chao1, Shannon and evenness in inferred microbiome patterns.

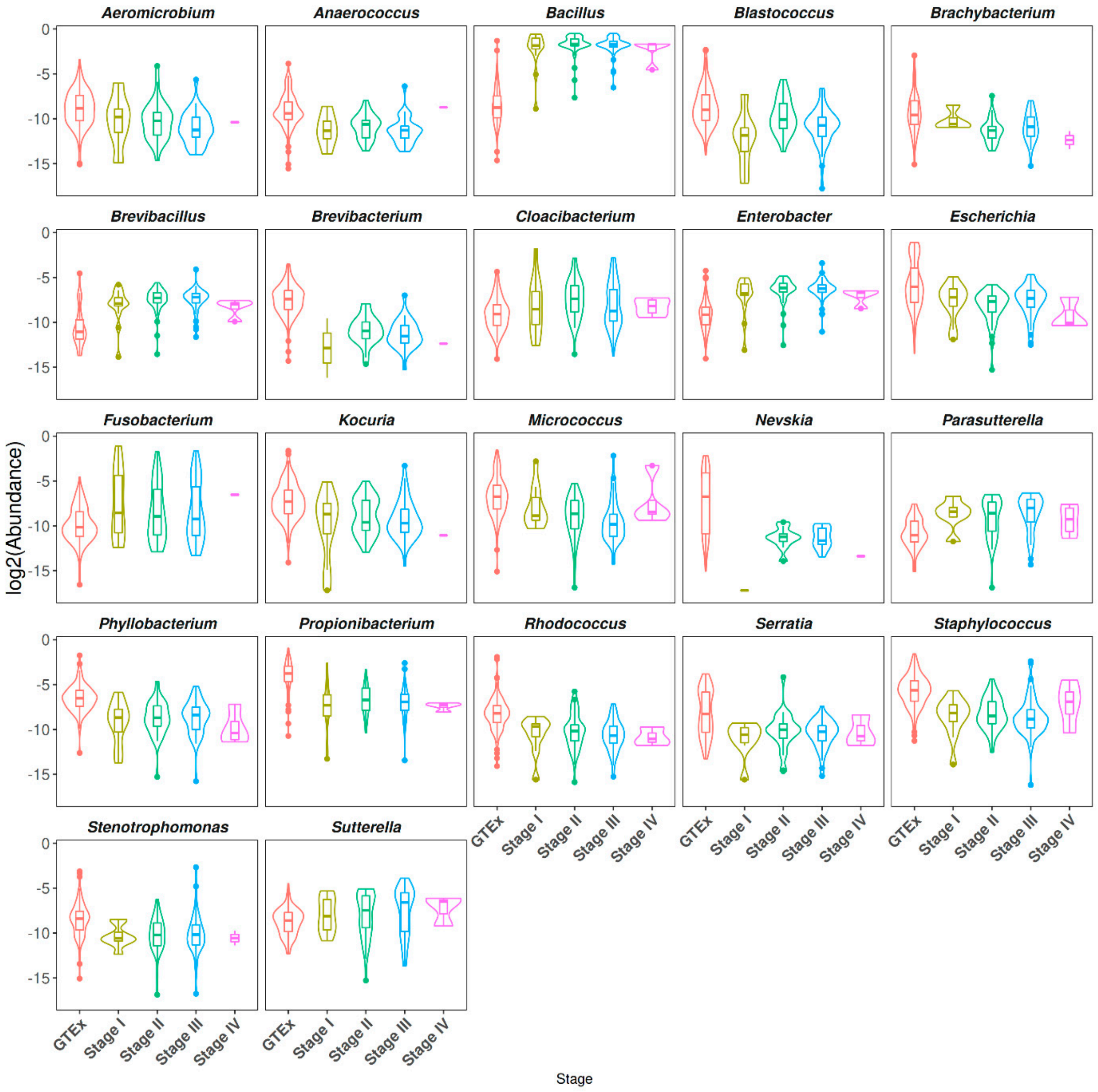

Supplementary Figure S4. Violin plot representation of the relative abundance for the statistically significant differentiated genera in the four cancer stages (AJCC pathological tumour stating) and GTEx non-cancer dataset.

References

1. Turnbaugh, P.J.; Ley, R.E.; Hamady, M.; Fraser-Liggett, C.M.; Knight, R.; Gordon, J.I. The human microbiome project. *Nature* **2007**, *449*, 804-810, doi:10.1038/nature06244.
2. Li, J.; Jia, H.; Cai, X.; Zhong, H.; Feng, Q.; Sunagawa, S.; Arumugam, M.; Kultima, J.R.; Prifti, E.; Nielsen, T., et al. An integrated catalog of reference genes in the human gut microbiome. *Nat Biotechnol* **2014**, *32*, 834-841, doi:10.1038/nbt.2942.
3. Rajilić-Stojanović, M.; de Vos, W.M. The first 1000 cultured species of the human gastrointestinal microbiota. *FEMS microbiology reviews* **2014**, *38*, 996-1047, doi:10.1111/1574-6976.12075.
4. Ferreira, R.M.; Pereira-Marques, J.; Pinto-Ribeiro, I.; Costa, J.L.; Carneiro, F.; Machado, J.C.; Figueiredo, C. Gastric microbial community profiling reveals a dysbiotic cancer-associated microbiota. *Gut* **2018**, *67*, 226-236, doi:10.1136/gutjnl-2017-314205.
5. Cavadas, B.; Ferreira, J.; Camacho, R.; Fonseca, N.A.; Pereira, L. QmihR: Pipeline for Quantification of Microbiome in Human RNA-seq. In Proceedings of International Conference on Practical Applications of Computational Biology & Bioinformatics; pp. 173-179.
